# Identification and characterization of wheat drought-responsive MYB transcription factors involved in the regulation of cuticle biosynthesis

**DOI:** 10.1093/jxb/erw298

**Published:** 2016-08-03

**Authors:** Huihui Bi, Sukanya Luang, Yuan Li, Natalia Bazanova, Sarah Morran, Zhihong Song, M. Ann Perera, Maria Hrmova, Nikolai Borisjuk, Sergiy Lopato

**Affiliations:** ^1^Australian Centre for Plant Functional Genomics, School of Agriculture, Food and Wine, University of Adelaide, Glen Osmond, South Australia 5064, Australia; ^2^W.M.Keck Metabolomics Research Laboratory, Iowa State University, Ames, IA 50011, USA

**Keywords:** Abiotic stress, cuticle, β-diketone, drought, molecular model, MYB and SHINE1 transcription factors, water deficit, wax, wheat.

## Abstract

A plant cuticle forms a hydrophobic layer covering plant organs, and plays an important role in plant development and protection from environmental stresses. We examined epicuticular structure, composition, and a MYB-based regulatory network in two Australian wheat cultivars, RAC875 and Kukri, with contrasting cuticle appearance (glaucousness) and drought tolerance. Metabolomics and microscopic analyses of epicuticular waxes revealed that the content of β-diketones was the major compositional and structural difference between RAC875 and Kukri. The content of β-diketones remained the same while those of alkanes and primary alcohols were increased by drought in both cultivars, suggesting that the interplay of all components rather than a single one defines the difference in drought tolerance between cultivars. Six wheat genes encoding MYB transcription factors (TFs) were cloned; four of them were regulated in flag leaves of both cultivars by rapid dehydration and/or slowly developing cyclic drought. The involvement of selected MYB TFs in the regulation of cuticle biosynthesis was confirmed by a transient expression assay in wheat cell culture, using the promoters of wheat genes encoding cuticle biosynthesis-related enzymes and the SHINE1 (SHN1) TF. Two functional MYB-responsive elements, specifically recognized by TaMYB74 but not by other MYB TFs, were localized in the *TdSHN1* promoter. Protein structural determinants underlying the binding specificity of TaMYB74 for functional DNA *cis*-elements were defined, using 3D protein molecular modelling. A scheme, linking drought-induced expression of the investigated TFs with downstream genes that participate in the synthesis of cuticle components, is proposed.

## Highlight

We uncovered significant structural and compositional differences between leaf cuticles of drought-tolerant and drought-sensitive wheat cultivars. Specific MYB factors drive cuticular responses to drought and may underlie genotypes with contrasting drought tolerance.

## Introduction

Wheat production is highly sensitive to environmental and climatic variation. Crops are often subjected to the negative influences of abiotic stresses, such as limited water supply, high salinity, and heat; these significantly impair grain yields (Porter and Semenov, 2005). The worst impact of temperature increases created by global warming is predicted to occur at low latitudes, where ~100 Mha of wheat are cultivated. These territories include the major wheat-cropping regions of Southern and Western Australia (Lobell and Gourdji, 2012). There is a growing consensus that combatting potential yield losses associated with these challenges could be achieved through selection and adaptation of cultivars with improved genetic potential (Reynolds *et al.*, 2012). Understanding the biochemical and molecular mechanisms which allow plants to cope with environmental challenges has a vital significance for improvement of stress tolerance and yield (Hrmova and Lopato, 2014). Towards this aim, two Australian wheat cultivars, Kukri and RAC875, both with excellent performance and superior grain quality, but showing contrasting drought tolerance (RAC875 outyielding Kukri by 24% under cyclic drought), have been subjected to intensive physiological (Izanloo *et al.*, 2008), genetic (Bennett *et al.*, 2012*a*, *b*), and metabolomic (Bowne *et al.*, 2012) investigations. These studies revealed a stronger ability of RAC875 to retain tissue water potential under drought; this trait may be linked to the glaucous appearance (glaucousness) of RAC875 compared with Kukri. Glaucousness is a bluish-white coloration of plant organs, and results from a visual reflection of light by certain epicuticular waxes accumulated in the form of wax crystals on plant surfaces.

The cuticle covers all plant aerial organs and provides protection during plant development and under biotic and abiotic stresses. It is composed of a cutin polyester layer, which is impregnated and covered with waxes composed of various aliphatic carbohydrates (Beisson *et al.*, 2012). Increased amounts of cuticular waxes are associated with improved drought tolerance in several different species (Borisjuk *et al.*, 2014; Baldoni *et al.*, 2015). Breeding for enhanced tolerance and performance under drought can sometimes lead to increased amounts of cuticular waxes (González and Ayerbe, 2010). However, it was found that wax composition is more important than total wax load for the formation of glaucousness in wheat (Zhang *et al.*, 2013). Further points on the role of the cuticle can be found in the Supplementary Introduction at *JXB* online.

Many Arabidopsis genes have been identified to be responsible for cuticular wax biosynthesis, transport, and accumulation (Beisson *et al.*, 2003; Jetter *et al.*, 2007; Jetter and Kunst, 2008; Li-Beisson *et al.*, 2013). Members of several families of transcription factors (TFs) are involved in the regulation of these genes. Most belong to one of three plant TF families: ethylene-responsive factors (ERFs), the myeloblastosis (MYB) family TFs, and homeodomain-leucine zipper class IV (HD-Zip IV) factors (Borisjuk *et al.*, 2014). Overexpression of these TFs alters cuticle deposition and/or composition, and often increases stress tolerance in transgenic plants (Aharoni *et al.*, 2004; Zhang *et al.*, 2005, 2007; Javelle *et al.*, 2010; Seo and Park, 2011; Seo *et al.*, 2011).

MYB TFs comprise one of the largest TF families and are involved in controlling various processes, including responses to biotic and abiotic stresses, development, differentiation, and metabolism (Ambawat *et al.*, 2013; Baldoni *et al.*, 2015). The MYB TF family is subdivided into four subfamilies according to the number of imperfectly repeated R1–R3 DNA-binding domains: 4R-MYB, 3R-MYB (R1R2R3-MYB), 1R-MYB or MYB-related proteins, and R2R3-MYB, which is the largest subfamily of MYB TFs in plants. The residues in the R1–R3 domains contribute to the correct formation of α-helices of MYB TFs and are required for specific base recognition of DNA (Oda *et al.*, 1997). All reported MYB-type regulators of cuticle biosynthesis belong to the R2R3-MYB subfamily of TFs; they are represented by AtMYB41, AtMYB16, AtMYB106, AtMYB96, and AtMYB30 from Arabidopsis, and SlMYB12 from tomato (Cominelli *et al.*, 2008; Raffaele *et al.*, 2008; Adato *et al.*, 2009; Gilding and Marks, 2010; Seo *et al.*, 2011; Oshima *et al.*, 2013).

The *AtMYB41* gene has a low level of expression in all analysed organs of Arabidopsis in the absence of stress, but it is strongly induced by abscisic acid (ABA), drought, and high salinity (Cominelli *et al.*, 2008). Overexpression of *AtMYB41* in transgenic Arabidopsis leads to an increased cuticle permeability. The expression of a number of genes related to lipid biosynthesis and transport, cuticle metabolism, and cell wall biosynthesis were found to be affected by overexpression of *AtMYB41* in transgenic Arabidopsis (Cominelli *et al.*, 2008).

Two other cuticle-related *MYB* genes, *AtMYB16* and *AtMYB106* [also known as *NOK*; the name originates from the Arabidopsis mutant *noeck* (*nok*)], are paralogous genes, involved in the formation of epidermal cell shape and regulation of cuticle biosynthesis by co-operating with the *WAX INDUCER1/SHINE1 (WIN1*/*SHN1*) gene in *Arabidopsis thaliana* and *Torenia fournieri* (Folkers *et al.*, 1997; Jakoby *et al.*, 2008; Gilding and Marks, 2010; Oshima *et al.*, 2013). Expression in transgenic Arabidopsis of AtMYB106 fused to a repressor domain (Hiratsu *et al.*, 2003), as well as knockout/knockdown of the *AtMYB106* and *AtMYB16* genes using RNAi, negatively influenced the formation of cuticle and resulted in the adhesion of flowering organs (Oshima and Mitsuda, 2013; Oshima *et al.*, 2013).

The Arabidopsis *MYB96* gene was initially identified as a regulator of drought stress responses of plants by integrating ABA and auxin signals; expression of *AtMYB96* was induced by ABA, drought, and high salinity. Constitutive overexpression of *AtMYB96* conferred drought tolerance to transgenic Arabidopsis, while a knockout mutant was more sensitive to drought than wild-type plants (Seo *et al.*, 2009). This was confirmed by studies of the loss-of-function mutant *myb96*, which also had sensitivity to drought (Guo *et al.*, 2013). Transcriptional activation by AtMYB96 of cuticular wax biosynthesis in connection with increased drought tolerance was originally reported by Seo *et al.* (2011). In that study, microarray analysis revealed that AtMYB96 activates a group of genes encoding cuticular wax biosynthetic enzymes, including several enzymes responsible for condensing of very long chain fatty acids (VLCFAs). Cuticular wax depositions in both leaves and stems were significantly increased in the activation-tagged *myb96-1D* mutant and decreased in the loss-of-function *myb96-1* mutant. The MYB recognition *cis*-element (TAACTA/G) was found in the promoters of target genes, and a direct interaction of AtMYB96 with promoters of genes encoding wax biosynthetic enzymes was demonstrated (Seo *et al.*, 2011). Strong constitutive expression of the Arabidopsis gene *AtMYB96* has been used to improve drought tolerance of an emerging oilseed crop plant, *Camelina sativa* (Lee *et al.*, 2014).

Two genes closely related to AtMYB96, AtMYB30 and AtMYB94, were shown to regulate cuticular wax biosynthetic genes (Raffaele *et al.*, 2008; Lee and Suh, 2015*a*). Among the putative AtMYB30 targets, genes were found encoding the four enzymes forming the acyl-coA elongase complex, which are responsible for the synthesis of VLCFAs (Raffaele *et al.*, 2008). Involvement of AtMYB94 in cuticular wax biosynthesis was confirmed by analysis of transgenic Arabidopsis with constitutive overexpression of this gene. A comparison of transgenic and control plants revealed enhanced expression of cuticular wax biosynthesis genes, increased accumulation of cuticular waxes, and a reduced rate of cuticular transpiration in transgenic plants (Lee and Suh, 2015*a*). It was shown that AtMYB94 activates the expression of wax biosynthetic genes *WSD1*, *KCS2/DAISY*, *CER2*, *FAR3*, and *ECR* by binding directly to their promoters (Lee and Suh, 2015*b*). The level of expression of the *AtMYB94* gene under drought was increased ~9-fold. An increased accumulation of cuticular waxes reduced the rate of cuticular transpiration in the leaves of *AtMYB94* transgenic Arabidopsis lines under drought (Lee and Suh, 2015*b*). Analysis of the *fused leaves 1* (*fdl1-1*) mutation in maize revealed involvement of the *Fdl1* gene product, ZmMYB94, in the regulation of cuticle deposition in young seedlings and the establishment of a regular pattern of epicuticular wax deposition on the epidermis of young leaves. Lack of *Fdl1* led to developmental defects (La Rocca *et al.*, 2015).

Another MYB TF, which is involved in plant cuticle regulation, is SlMYB12. Detailed gene expression and metabolomics analyses of transgenic tomato plants revealed involvement of SlMYB12 in regulation of tomato fruit cuticle biosynthesis (Adato *et al.*, 2009). The Arabidopsis homologue of this gene has not been characterized.

In this study, we investigated the biochemical background of cuticular waxes in two wheat cultivars, RAC875 and Kukri, grown under well-watered conditions and mild drought. We identified and isolated six *MYB* genes from RAC875, encoding homologues of known cuticle biosynthesis-related Arabidopsis and tomato MYB TFs, and characterized for their involvement in the regulation of cuticle formation in wheat under water deficit.

## Materials and methods

### Plant material and cultivation

Wheat plants *Triticum aestivum*, cultivars RAC875 and Kukri, previously described by Izanloo *et al.* (2008), were grown in a greenhouse in 112×76×50cm containers, equipped with an automatic watering system and continuous monitoring of the soil water potential (Amalraj *et al.*, 2016). For the cyclic drought experiment, drought-tolerant RAC875 and drought-sensitive Kukri were grown as previously described (Harris *et al.*, 2016). A drought treatment was applied to half of the plants, according to a scheme adopted by Bowne *et al.* (2012) and depicted in Supplementary Fig. S1. Watering in the first cycle of drought was withdrawn at flag leaf emergence until the drought-sensitive Kukri showed wilting. Plants were then re-watered to field capacity and again left to dry without watering until Kukri once again reached wilting point. In this experiment, re-watering was done twice: at 15 d and 24 d after flag leaf emergence and the initial withholding of watering (Supplementary Fig. S1). Plant water status was monitored for both cultivars by measuring the relative water content of the detached second leaf, as was described by Izanloo *et al.* (2008).

### Cloning of the wheat orthologues of selected MYB TFs

Amino acid and/or nucleotide sequences of selected MYBs (AtMYB41, AtMYB96, AtMYB106, AtMYB16, and SlMYB12) were retrieved from the National Center for Biotechnology Information (NCBI, Bethesda, MD, USA) or the Arabidopsis Information Resource (TAIR, Columbus, OH, USA) databases, using the accession and/or locus numbers summarized by Borisjuk *et al.* (2014). Retrieved sequences were used to search against the latest versions of the wheat genomic and cDNA sequence databases linked to the Blast Portal at the Australian Centre for Plant Functional Genomics (ACPFG, University of Adelaide, Australia) to ensure that the closest wheat genes were identified. The identified wheat sequences were used to design primers (Supplementary Table S1) for gene amplification by nested PCR from cDNA pools prepared from the leaves and spikes of the drought-tolerant wheat cultivar RAC875 subjected to drought. CACC sequences were added to the 5' ends of the forward primers used in the second round of PCR to conduct directional cloning of full-length coding sequences (CDS) of each gene into the pENTR/D-TOPO vector (Life Technologies, Victoria, Australia).

### Gene expression analysis in different wheat tissues, under dehydration and cyclic drought

Gene expression of selected MYB genes was investigated in detached leaves subjected to rapid dehydration, and in plants subjected to cyclic drought (described above). To analyse the response of genes to rapid dehydration, flag leaves were cut from four well-watered plants of each of the cultivars RAC875 and Kukri at awn emergence. Leaves were placed in 12ml open plastic test tubes, incubated at ambient temperature (23 °C) for 0, 2, 4, and 7h, then frozen in liquid nitrogen and stored at −80 °C for RNA extraction. Flag leaf samples were also collected from both cultivars during the cyclic drought experiment, at 5, 9, 14, 23, and 25 d after initiating the first cycle of drought (Supplementary Fig. S1). A set of samples was collected from well-watered (control) plants at the same time points. Total RNA was isolated from leaf tissues using a Direct-zol RNA MiniPrep Kit (Zymo Research, CA, USA) with an on-column DNase treatment. A 1.5 μg aliquot of purified RNA from each sample was used for cDNA synthesis using a SuperScript III Reverse Transcriptase kit (Life Technologies, Victoria, Australia). Quantitative real-time PCR (Q-PCR) analysis was performed on cDNA samples as described previously (Fletcher, 2014). Three wheat genes, encoding actin, cyclophilin, elongation α factor, and glyceraldehyde-3-phosphate dehydrogenase, were simultaneously used for normalization of expression (Fletcher, 2014). The selection of three genes was based on the pairwise comparison among the three genes mentioned above. To obtain the actual copy numbers of RNA, we generated a standard curve of the copy number in relation to the cycle threshold (Ct) value. The standard curve was constructed using a dilution series, prepared from the purified PCR product of the target gene, covering six orders of magnitudes. To analyse tissue specificity of a selected subset of the genes, we also utilized a cDNA series prepared from different tissues of *T. aestivum* cv. Chinese Spring (Morran *et al.*, 2011). Three biological and three technical replicates were used in all gene expression analysis experiments.

### In-yeast activation assays and localization of activation domains

Sets of full-length and partial CDS for *TaMYB16*, *TaMYB24*, *TaMYB31*, *TaMYB74*, *TaMYB77*, and *TaMYB78* were amplified by PCR with *Eco*RI and *Bam*HI restriction sites introduced in forward and reverse primers, respectively, and cloned in the same restriction sites of the pGBKT7 vector (Scientifix, Victoria, Australia). Each set included the full-length CDS, and versions with truncations at the 3' end. A transcriptional activation assay was performed as previously described by Eini *et al.* (2013). Generated constructs were transformed into yeast (*Saccharomyces cerevisiae*) strain Y187 as described by Pyvovarenko and Lopato (2011). The pGBKT7 vector harbours a tryptophan (Trp) selection gene. The yeast reporter strain, Y187, could not grow on the synthetic defined (SD) medium lacking Trp without introducing a functional *TRP1* gene and could not grow on the SD/-His medium without activation of a *HIS3* gene. Therefore, yeast transformants were first selected on the SD/-Trp medium to prove that transformation of the pGBKT7 construct in yeast cells occurred. The yeast culture was replica-plated onto the SD/-Trp/-His medium. The ability of full-length or truncated wheat MYB proteins to activate expression of the *HIS3* gene led to yeast growth on the selective medium.

### Assessment of promoter activation by MYB TFs in a wheat transient expression assay

A transient expression assay was performed using *Triticum monoccocum* L. suspension cell culture, according to the procedure established by Eini *et al.* (2013). In this assay, cultivated wheat cells were co-bombarded with vectors expressing one of the *MYB* TF genes in a pair with a construct containing the β-glucuronidase reporter gene (*GUS*) fused to a promoter with potential MYB-binding sites. GUS expression from the MYB-activated promoter was quantified 48h after bombardment. Promoters of three cuticle biosynthesis-related genes: 3-ketoacyl CoA synthetase (*KCS1*), cytochrome P450 monooxygenase (*ATT1*), and transcription factor *SHN1* (Borisjuk *et al.*, 2014), were selected as targets for activation by MYB TFs. The promoter sequences of the *TaKCS1* and *TaATT1* genes (3235bp and 2535bp fragments upstream of the corresponding gene translational sites) were cloned by nested PCR, using primers based on corresponding gene sequences derived from the International Wheat Genome Sequencing Consortium (IWGSC; http://www.wheatgenome.org/) databases, and genomic DNA of *T. aestivum* cv. RAC875 as template. The sequence of the *SHN1* promoter was obtained using a clone from a BAC (bacterial artificial chromosome) library of *Triticum durum* cv. Langdon (Cenci *et al.*, 2003). The full-length CDS of *TaSHN1* (624bp) was isolated by PCR and used to screen the BAC library by colony hybridization. The plasmid of the selected BAC clone was isolated using a Large Construct Kit (QIAGEN, Hilden, Germany), and the presence of the *TdSHN1* gene was confirmed by PCR using the primers listed in Supplementary Table S1; the BAC clone was sequenced using 454 sequencing technology (Wicker *et al.*, 2006). The obtained sequence was used to design primers and amplify a 2203bp fragment of the *TdSHN1* promoter. The three promoters, as well as six 5'-deletion variants of the *TdSHN1* promoter, were cloned into the pENTR-D-TOPO vector (Life Technologies, Victoria, Australia) and re-cloned by recombination upstream of the *GUS* gene into the expression vector pMDC164 (Curtis and Grossniklaus, 2003). Vectors for expression of MYB proteins were constructed by recombinational cloning of the *TaMYB24*, *TaMYB31*, *TaMYB74*, and *TaMYB77* CDS into the modified pMDC32 vector (Curtis and Grossniklaus, 2003), where the standard 35S promoter was replaced with a maize polyubiquitin promoter, pUbi (Christensen *et al.*, 1992). The pUbi–green fluorescent protein (GFP) construct was generated in a similar way, by cloning of CDS encoding a GFP in the same vector, and it was used as a negative control in all transient expression experiments. MYB recognition (MYBR) *cis*-elements were predicted using the Plant *Cis*-acting Regulatory DNA Elements database (PLACE, University of Pittsburgh, USA) (Higo *et al.*, 1999) prior to selection and cloning of promoter deletions.

### Composition analysis of cuticular waxes

For the wax composition analysis, 6.5cm long flag leaf segments were collected at 24 d after anthesis. The weight of each leaf was measured, and leaves were immersed in liquid nitrogen for storage at −80 °C. For wax extraction, frozen leaf samples were warmed to ambient temperature for 2min. Hexadecane (C16 alkane), used as an internal standard, was dissolved in hexane and applied to the surfaces of leaves in amounts of 1 µg per 0.3g of a leaf sample. At 3–5min after application of the internal standard, waxes were extracted by dipping into 4ml of chloroform for 1min and dried under a stream of nitrogen. GC-MS analysis was conducted in the W.M. Keck Metabolomics Research Laboratory of Iowa State University (USA). The wax extract was dissolved in 200 μl of acetonitrile, spiked with 1 μg of triacontane (dissolved in chloroform), and derivatized with 50 μl of *N*,*O*-bis(trimethylsilyl)trifluoroacetamide with 1% (v/v) trimethylchlorosilane at 80 °C for 60min. The sample was dried under a stream of nitrogen; the residue was reconstituted in 100 μl of chloroform and subjected to GC-MS analysis according to the procedure described in Cha *et al.* (2009). GC-MS analysis was performed with an Agilent 6890 GC (Agilent Technologies, CA, USA) interfaced to a 5973 mass spectrometer. The HP-5ms column (30 m×0.25 mm×0.25 μm) was used and a temperature gradient was programmed from 120 °C to 325 °C at 5 °C min^–1^ with a He flow rate at 1.0ml min^–1^. Operating parameters for MS were set to 70eV of ionization voltage and 280 °C of interface temperature. The GC-MS data files were de-convoluted by the NIST AMDIS software and searched using an in-house compound library and the NIST 2014 Mass Spectral Library.

### Scanning electron microscopy

The epicuticular wax structure was examined using a scanning electron microscope in the Adelaide Microscopy Unit (https://www.adelaide.edu.au/microscopy/, University of Adelaide, Australia). All analyses of wheat cuticular waxes were performed using flag leaves, the main source of assimilates during grain development (Evans *et al.*, 1975), and the standard subject for cuticle analysis in wheat (Adamski *et al.*, 2013; Zhang *et al.*, 2013). Flag leaf blades were collected 10 d after anthesis, and segments close to the major vein of ~0.4×0.3cm in size were cut from the middle of leaves and examined under a Philips XL30 Field Emission Scanning Electron Microscope, equipped with a Gatan CT1500 HF Cryo-transfer Stage. Samples were attached to the holder using Tissue-Tek OCT compound mixed with carbon dag in 1:1 ratio (carbon dag is a commonly used name for the conductive carbon paint), after which they were frozen in liquid nitrogen, and transferred under vacuum to the preparation chamber. The temperature of samples was raised to −92 °C, and held for ~2min, to allow ice on the surface to sublime away. The temperature was lowered to −110 °C (at which sublimation ceased), and the sample was coated with platinum (~2nm thick layer) to make it electrically conductive. The sample was loaded onto the microscope stage (held at a temperature lower than −150 °C) and examined.

### Simulation of evolutionary relationships of MYB proteins

To construct a phylogenetic tree of MYB factors (Fig. 2), we used 103 wheat sequences from the Plant Transcription Factor Database (http://planttfdb.cbi.pku.edu.cn/, Center for Bioinformatics, Peking University, China), 27 wheat R2R3 MYB gene sequences identified by Zhang *et al.* (2012), together with six wheat genes characterized in this study, and five MYB query genes from Arabidopsis and tomato. The evolutionary history of representative MYB proteins was inferred using the Neighbor–Joining method (Saitou and Nei, 1987). Evolutionary distances were computed using the p-distance method (Nei and Kumar, 2000) (with 1000 bootstrap replications), and expressed in units of numbers of residue differences per site. All positions containing gaps and missing data were eliminated. Evolutionary analyses were conducted in MEGA6 (Tamura *et al.*, 2013).

### 3D protein molecular modelling

Homology modelling of TaMYB74 was performed with Modeller v9.10 (Eswar *et al.*, 2008). The TvMYB2 protein structure from the protozoan parasite *Trichomonas vaginalis* in complex with MRE-1–12 DNA (5'-AAATATCGTTAT-3'/5'-ATAACGATATTT-3') (Protein Data Bank accession 3OSG) (Jiang *et al.*, 2011) was used as a structural template. The primary sequence of TaMYB74 shares 34.4% identity and 45.6% similarity with the TvMYB2 protein. Selected models displaying the lowest objective function values were analysed by ProSA2003 (Sippl, 1993) and PROCHECK to evaluate stereochemical and G-factor properties (Laskowski *et al.*, 1993). The target DNA *cis*-element for TaMYB74 (denoted as MYBR1: 5'-AGGTGGTTATGC-3'/5'-GCATAACCACCT-3'; the core sequence is underlined) was generated based on the DNA structure of the MRE-1–12 *cis*-element using Coot (Emsley *et al.*, 2010). The most favourable TaMYB74 structural model with a DNA *cis*-element was minimized in YASARA (Krieger *et al.*, 2009) and evaluated. The Ramachandran plot of the DNA–TF complex structure showed that 100% of residues were located in the most favoured and additional allowed regions, with an overall G-factor of −0.17 (PROCHECK) and a *z*-score value of −5.9 (ProSa2003). The overall G-factor for the TvMYB2–DNA structure is 0.24 and the *z*-score value is −7.6. Energy (or conformational stability) analyses were calculated using Fold-X force-field (Schymkowitz *et al.*, 2005).

### Statistical analysis of data

Quantification data of wax components and data on gene expression levels under dehydration and cyclic drought were analysed using two-way ANOVA with the Fisher’s least significant difference post-hoc test. Transient expression assay data were analysed using one-way ANOVA with the Fisher’s least significant difference post-hoc test. All analyses were conducted in GenStat (16th Edition; VSN International Ltd, Hemel Hempstead, UK).

### GenBank accession numbers

*TaMYB24*, KU674896; *TaMYB31*, KU674897; *TaMYB74*, KU674898; *TaMYB16*, KU674899; *TaMYB77*, KU67900; *TaMYB78*, KU67901; *TaSHN1*, KU737577; *TaATT1* promoter, KU737578; *TaKCS1* promoter, KU737579; *TdSHN1* promoter, KU737580.

## Results

### Microscopic and biochemical characterization of cuticular waxes of Kukri and RAC875 cultivars grown under well-watered and mild drought conditions

**Fig. 1.**
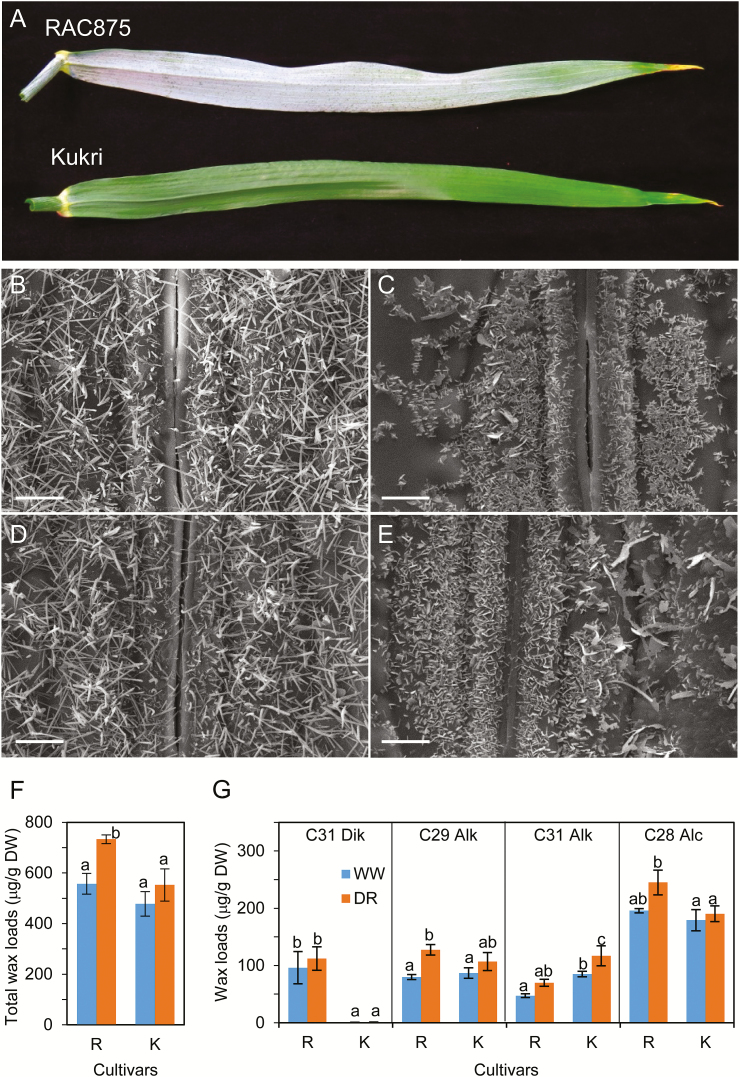
The visual appearance, ultrastructure, and the wax composition of cuticle on flag leaves of wheat. (A) The appearance of abaxial sides of flag leaves detached from RAC875 and Kukri wheat cultivars grown under well-watered conditions. (B and C) Scanning electron micrographs of the abaxial sides of flag leaves derived from RAC875 and Kukri plants grown under well-watered conditions. (D and E) Scanning electron micrographs of the abaxial side of leaves derived from RAC875 and Kukri plants grown under the conditions of limited watering (mild drought). (F and G) Total wax loads and amounts of the four most abundant wax components on the flag leaves of RAC875 (R) and Kukri (K) under well-watered (WW) and mild drought (DR) conditions. Wax loads were calculated as µg of wax per dry leaf weight (DW). Dik, C31 β-diketones; Alk, alkane; Alc, primary alcohol. Means and SEs were calculated from three replicates. Two-way ANOVA with the Fisher’s least significant difference post-hoc test was conducted using GenStat. The same lower case letters on top of error bars indicate differences that are not significant at the 5% level. Scale bars=5 µm.

Two Australian wheat cultivars, Kukri and RAC875, have contrasting drought and heat tolerance and have been intensively studied at physiological, genetic, and metabolomic levels (Izanloo *et al.*, 2008; Bennett *et al.*, 2012*b*; Bowne *et al.*, 2012). The results of these studies suggest a link between differences in water-retaining capacity and glaucousness. RAC875 has glaucous leaves and is drought tolerant, while Kukri has a non-glaucous phenotype (Fig. 1A). We applied SEM and GC-MS to compare the wax crystal structure and biochemical make-up of leaf blade surfaces in Kukri and RAC875, grown under well-watered and mild drought conditions. Under both well-watered (Fig. 1B) and mild drought conditions (Fig. 1D), tubule-shaped crystals, which have been suggested to result from the high content of β-diketones (Adamski *et al.*, 2013; Zhang *et al.*, 2013), were abundant on the abaxial side of the RAC875 flag leaves. The same surfaces in Kukri had platelet-shaped wax crystals (Fig. 1C, E), suggestive of a high content of primary alcohols (von Wettstein-Knowles, 2012). Under drought conditions, the number of wax crystals on the abaxial side of RAC875 leaves remains unchanged, while the number of those on the abaxial side of the Kukri leaves was slightly increased. However, no changes in crystal shapes were observed (Fig. 1D, E).

To reveal the chemical basis underlying the substantial differences in shape of wax crystals of these two cultivars, we performed GC-MS compositional analysis of extracted wax (Fig. 1F, G; Supplementary Fig. S2). There was a significant increase in total wax loads on leaves of plants grown under drought conditions compared with well-watered plants in RAC875, while a small but definite increase in Kukri was also observed (Fig. 1F). The main difference between the RAC875 and Kukri wax components was the presence of β-diketones in the wax of RAC875. β-Diketones were estimated to comprise ~18% of total waxes in RAC875, but were almost undetectable in Kukri (Fig. 1G). The increase in total wax loads was predominantly defined by an elevated accumulation of alkanes with a chain length of 29 and 31 carbons in both cultivars, and also of primary alcohols in RAC875 with the dominant chain length of 28 carbons (Fig. 1G). No significant difference was observed in the content of β-diketones in RAC875 between well-watered and mild drought conditions.

### Gene cloning and the phylogenetic relationships of MYB TFs

**Fig. 2.**
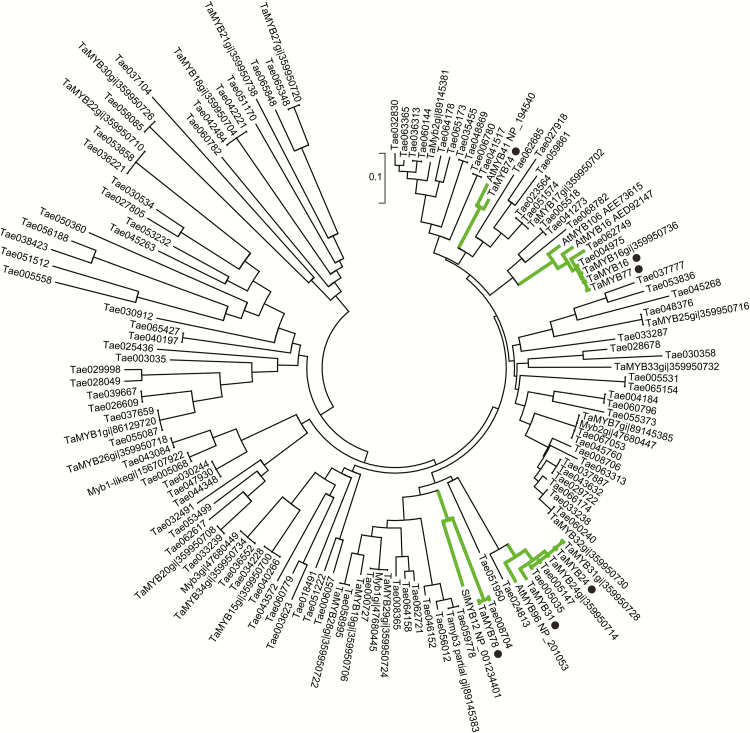
A phylogenetic tree of MYB TFs. We analysed 141 sequences including six sequences of wheat MYB TFs (indicated by dots) derived from cDNAs cloned in this work, five query Arabidopsis and tomato MYB TFs (GenBank accessions shown in the figure), 103 wheat MYB factors from the Plant Transcription Factor Database (annotated as Tae with a six-figure number) and 27 wheat R2R3 MYB TFs (Zhang *et al.*, 2012). The branches, to which wheat MYB protein sequences studied in this work belong, are indicated with thick grey lines. The number near a scale indicates a residue difference per site. The tree was constructed using the Neighbour–Joining method in MEGA6. (This figure is available in colour at *JXB* online.)

Six wheat *MYB* genes were cloned by nested PCR from leaves and spikes of the drought-tolerant wheat cultivar RAC875. The protein sequences of five known cuticle regulators from Arabidopsis and tomato, AtMYB41, AtMYB16, AtMYB106, AtMYB96, and SlMYB12, were used to identify protein and nucleotide sequences of the closest wheat homologues in several wheat databases. Details of the six cloned wheat *MYB* genes, including their names, accession numbers, corresponding Arabidopsis homologues, and their proposed chromosomal locations are summarized in Table 1. Schematic representations of gene structures of the six wheat genes, using the Gene Structure Display Server (GSDS 2.0), are shown in Supplementary Fig. S3. The evolutionary relationship of 141 members, which include four TFs from Arabidopsis and a sequence from tomato together with six wheat homologues/orthologues, as well as 103 wheat MYB sequences from the Plant Transcription Factor Database and 27 wheat R2R3 MYB sequences previously identified by Zhang *et al.* (2012), was inferred by using the Neighbor–Joining method in MEGA6 (Fig. 2). The phylogenetic tree shows that all cloned wheat sequences (indicated by dots in Fig. 2) cluster with their corresponding Arabidopsis protein homologues. The tree confirmed that six wheat and respective Arabidopsis MYB sequences identified in this work are related. Further points on gene cloning and the phylogenetic relationships of MYB TFs can be found in the Supplementary Results.

**Table 1.** Cloned wheat MYB genes Gene locations on wheat chromosomes are based on *in silico* analysis using the International Wheat Genome Sequencing Consortium (IWGSC) database. References for each of the query genes are listed in the right column.

Cloned wheat genes	Accession numbers	Query genes	Coding sequence length (bp)	Genetic location	Query sequence references
*TaMYB16*	KU674899	*AtMYB106*	978	2DL	Oshima *et al.* (2013)
*TaMYB24*	KU674896	*AtMYB96*	945	2AS	Seo *et al.* (2009, 2011); Seo and Park (2010)
*TaMYB31*	KU674897	*AtMYB96*	954	5BL	Seo *et al.* (2009, 2011); Seo and Park (2010)
*TaMYB74*	KU674898	*AtMYB41*	1047	2DS	Cominelli *et al.* (2008)
*TaMYB77*	KU674900	*AtMYB16*	1059	2DL	Oshima *et al.* (2013)
*TaMYB78*	KU674901	*SlMYB12*	1041	4AS	Adato *et al.* (2009)

### Domain structure and the activation properties of cuticle-related MYB TFs

**Fig. 3.**
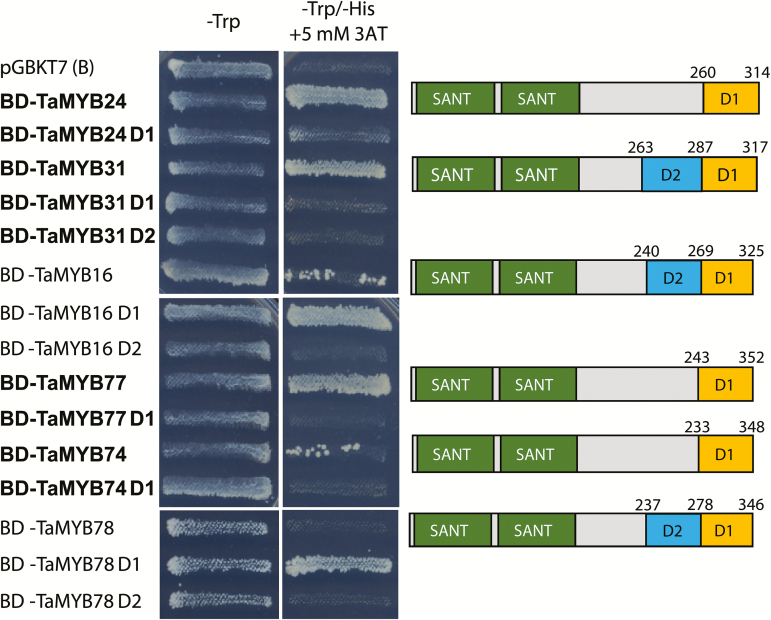
Transcriptional activation assays and the localization of activation domains of cloned *MYB* genes. The assay was performed in yeast using full-length and C-terminal truncated MYB TFs fused to a binding domain (BD) of yeast GAL4 TF. An empty pGBKT7 plasmid was used as a negative control. -Trp represents the synthetic defined (SD) medium lacking tryptophan (selection for plasmid presence) and -Trp/-His refers to the SD medium without tryptophan and histidine (selection for activation of the yeast *HIS3* gene). Drought-responsive MYBs and their truncations are shown in bold. Domain structures and positions of truncations are indicated in the right part of the figure. SANT: *S*wi3, *A*da2, *N*-Cor, and *T*FIIIB DNA-binding domains. D1 and D2 represent removed protein fragments; D2 truncation included the removal of D1. The residue positions of truncations are indicated. (This figure is available in colour at *JXB* online.)

Domain organization of the six MYB proteins, encoded by cloned cDNAs, was investigated using the SMART protein domain analysis server (Letunic *et al.*, 2015) (http://smart.embl-heidelberg.de/). Each of the wheat MYB TFs contains two adjacent highly conserved SANT DNA-binding domains, localized in the N-terminal part of the protein (Fig. 3), and represent characteristic features of plant R2 and R3 MYB TFs (Stracke *et al.*, 2001).

The presence and positions of activation domains (ADs) in six wheat MYB proteins were examined in yeast. For this purpose, full-length and truncated coding regions of the *MYB* genes were fused to the sequence encoding the binding domain of the yeast GAL4 TF. Constructs were used to transform yeast cells, and the presence of ADs in MYB TFs was revealed as the ability of the yeasts to grow on a selective medium. To obtain insights into the position and approximate length of the ADs of wheat MYB TFs, their amino acid sequences were truncated at the C-termini. The predictions of transcriptional ADs (under AD we mean here any sequence which is functionally important for the activation of transcription, rather than a conserved protein domain) in MYB proteins were based on knowledge that ADs are usually (but not always) enriched in acidic amino acid residues and contain glutamine-rich and proline-rich motifs (Johnson *et al.*, 1993).

Three full-length proteins, TaMYB24, TaMYB31, and TaMYB77, provided strong transcriptional activation, and one full-length protein, TaMYB74, resulted in weak transcriptional activation of the yeast *HIS3* gene, the product of which supports yeast growth on the selective medium deficient in histidine (Fig. 3). For the truncations, truncation D1 removed significant parts of predicted ADs in TaMYB24 and TaMYB31, and completely removed the ability of TaMYB77 and TaMYB74 to activate the reporter gene. Surprisingly, the full-length TaMYB16 showed weak activation of the *HIS3* gene and full-length TaMYB78 did not show any transcriptional activity in yeast. However, D1 removal in both proteins released their strong activator properties, suggesting either the presence of repressor motifs in D1, or changes in folding patterns of proteins. D2 removal, however, totally abolished the activation properties of TaMYB16 and TaMYB78, suggesting that the whole or the significant part of protein sequences responsible for transcriptional activation are located in D2.

### Selection of MYB genes that are regulated by water deficit

To identify cuticle biosynthesis-related regulatory genes responsive to water deficiency, expression of each of the cloned *MYB* genes was analysed by Q-PCR: (i) in detached flag leaves of RAC875 and Kukri that were subjected to rapid dehydration; and (ii) in flag leaves of the same two cultivars growing under cyclic drought.

**Fig. 4.**
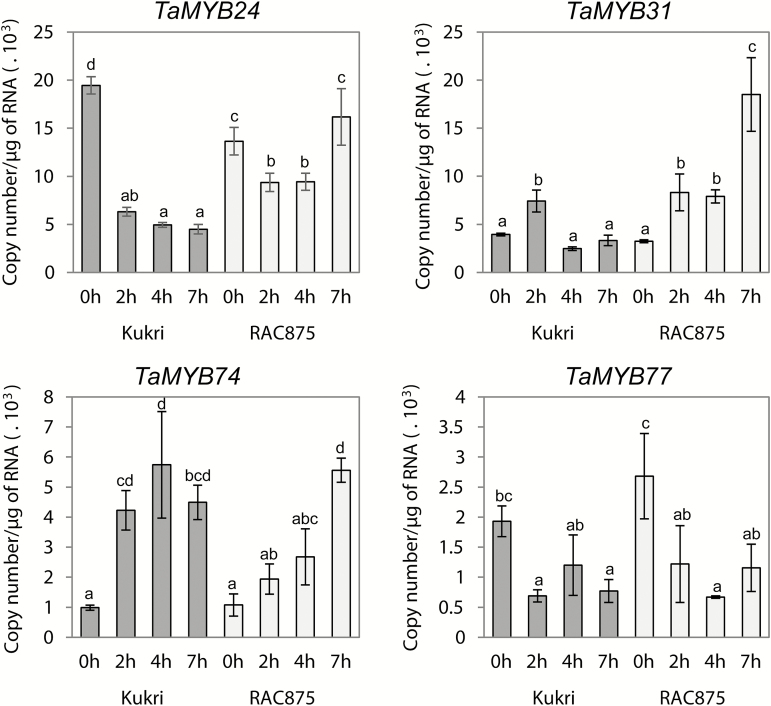
Expression levels of cloned *MYB* genes in rapidly dehydrating leaves of Kukri and RAC875. Expression of *TaMYB24*, *TaMYB31*, *TaMYB74*, and *TaMYB77* was studied by Q-PCR. Flag leaf samples were sampled at awn emergence. Dehydration was performed at room temperature for 0, 2, 4, and 7h, after which leaves were snap-frozen in liquid nitrogen. Two-way ANOVA with the Fisher’s least significant difference post-hoc test was conducted using GenStat. Error bars indicate the SE of three replicates.

**Fig. 5.**
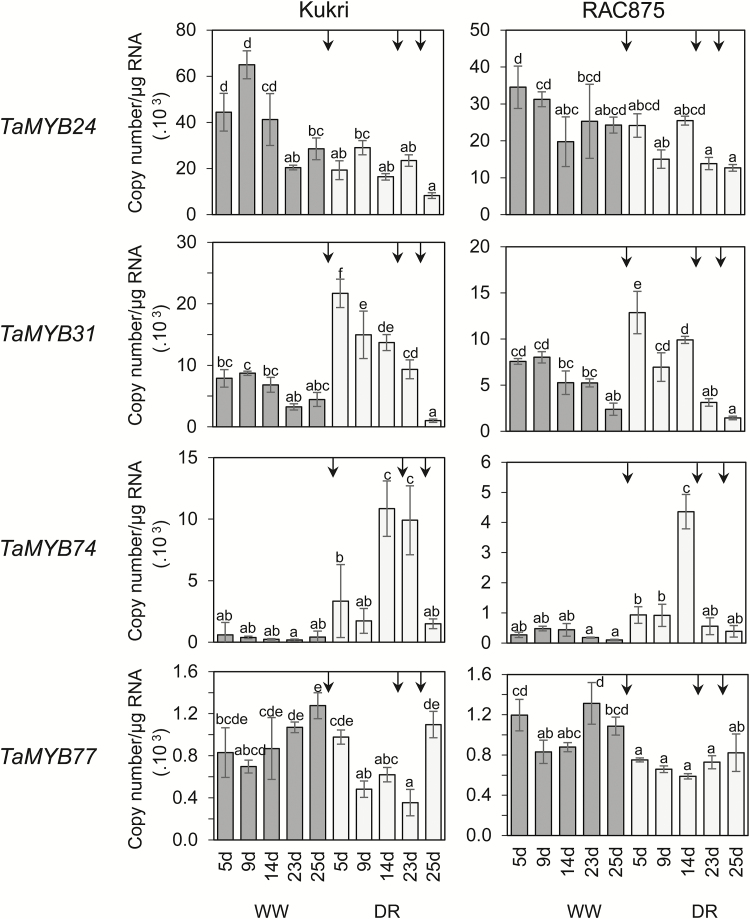
Expression levels of cloned *MYB* genes under cyclic drought in Kukri and RAC875. Expression of *TaMYB24*, *TaMYB31*, *TaMYB74*, and *TaMYB77* was studied by Q-PCR. Expression of genes was examined after 5, 9, 14, 23, and 25 d, using either well-watered (WW) or cyclic drought-exposed (DR) plants. Three cycles of drought (after watering points 1–3) indicated by arrows were applied at 0, 15, and 24 d as shown in Supplementary Fig. S1. Two-way ANOVA with the Fisher’s least significant difference post-hoc test was conducted using GenStat. Error bars indicate the SE of three replicates.

Two out of six examined genes, *TaMYB16* and *TaMYB78*, had no detectable gene expression in leaves during a rapid dehydration experiment (Fig. 4). Expression of these two *MYB* genes was not tested during the cyclic drought experiment because flag leaves of a similar developmental stage were used in both experiments. Of the remaining four genes, *TaMYB24* and *TaMYB77* were down-regulated, and *TaMYB31* and *TaMYB74* were up-regulated during rapid dehydration and under drought (Figs 4, 5). Induction of the expression of the *TaMYB24*, *TaMYB31*, *TaMYB74*, and *TaMYB77* genes was investigated in the flag leaves of wheat cultivars Kukri and RAC875 during three consecutive cycles of drought (Fig. 5). Water status during the cyclic drought experiment and the time points of leaf sampling are shown in Supplementary Fig. S1. Further points on the selection of *MYB* genes that are regulated by water deficit can be found in the Supplementary Results.

### Expression of MYB genes in wheat tissues

**Fig. 6.**
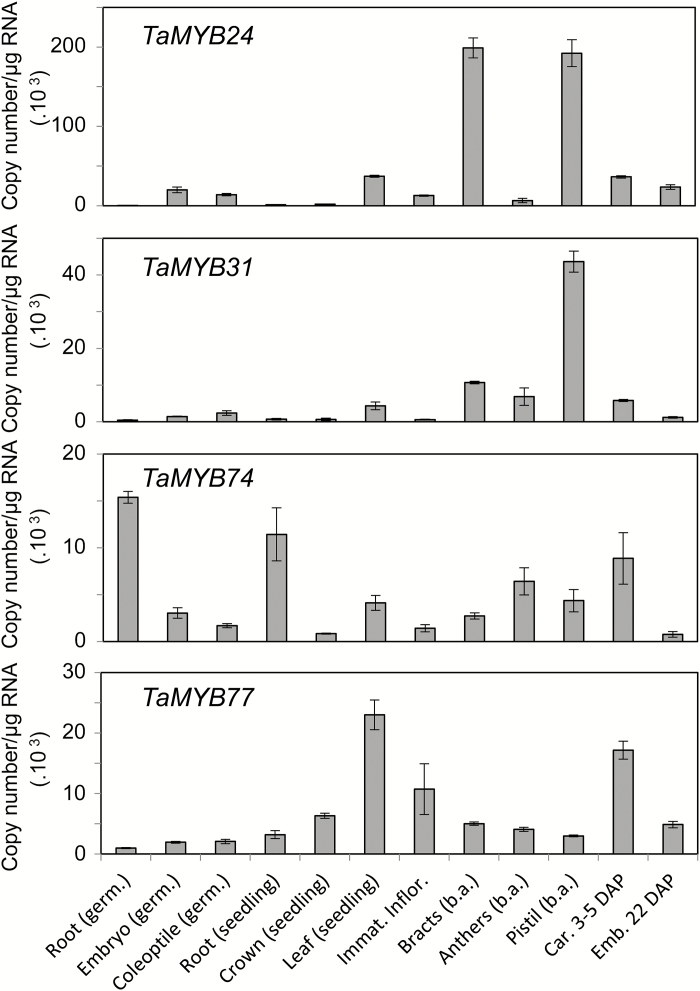
Expression profiles of *TaMYB24*, *TaMYB31*, *TaMYB74*, and *TaMYB77* in wheat tissues revealed by Q-PCR. germ., germinating seed; Emb., embryo; Immat. Inflor., immature inflorescence; b.a., before anthesis; Car., caryopsis; DAP, days after pollination. Error bars indicate the SE of three replicates.

Expression of dehydration- or drought-responsive *MYB* genes was tested in various tissues of *T. aestivum* cv. Chinese spring (Fig. 6). The *TaMYB24* gene showed the highest transcript levels in bracts and pistil before anthesis, and moderate levels in leaves and developing and mature caryopses. In all other tested tissues, the expression levels of *TaMYB24* were low. The expression pattern of the *TaMYB31* gene was similar to that of its homologue, *TaMYB24*, but overall the levels of expression were lower.

The highest expression level of the *TaMYB74* gene was found in roots. Two- to three-fold lower transcript levels were detected in leaves, anthers, pistil, and developing caryopsis.

The *TaMYB77* gene had the highest expression levels in leaf, developing caryopsis, and immature inflorescence. The transcript levels of this gene in other tested tissues were low.

### Activation of promoters of cuticle biosynthesis-related genes by drought-responsive MYB TFs

**Fig. 7.**
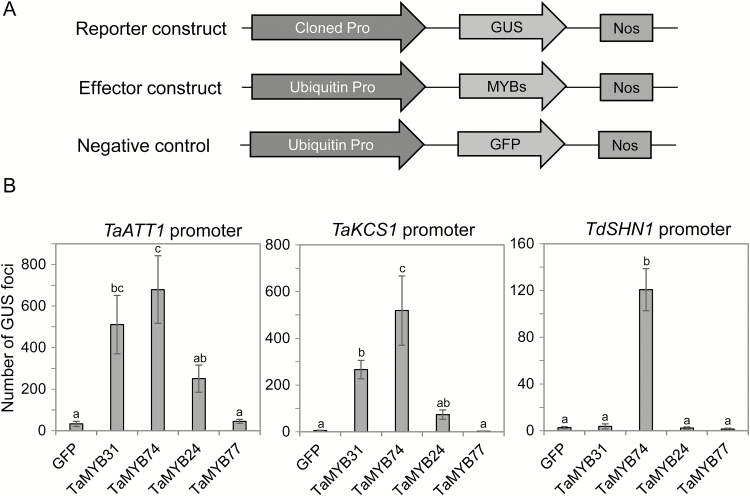
Activation of promoters of cuticle-related genes *TaATT1*, *TaKCS1*, and *TdSHN1* by drought-responsive MYB TFs. The data were obtained by a transient expression assay in a wheat suspension culture. (A) Schematic showing DNA constructs used in the transient expression assay. The reporter *GUS* gene was driven by one of three promoters of cuticle biosynthesis genes, *TaATT1*, *TaKCS1*, and *TdSHN1*. In effector constructs, wheat *MYB* genes were cloned under the control of the ubiquitin promoter. *GFP* served as a negative control. (B) Activation of *GUS* expression fused with promoters of *TaATT1*, *TaKCS1*, and *TdSHN1* by drought-responsive MYB factors. Each reporter construct was co-bombarded with each effector and *GFP* construct into a wheat suspension culture. One-way ANOVA with the Fisher’s least significant difference post-hoc test was conducted using GenStat. Error bars indicate the SE of three replicates.

Three promoters of cuticle biosynthesis-related genes were cloned either by nested PCR using genomic DNA of *T. aestivum* as template (*TaATT1* and *TaKCS1* promoters), or via screening of a BAC library of *T. durum* (*TdSHN1* promoter). Promoters were cloned upstream of the *GUS* reporter gene and the resulting constructs were used in transient expression assays. These assays were conducted to confirm the involvement of cloned *MYB* genes in the regulation of cuticle biosynthesis. Transient expression assays were performed by co-bombardment of a suspension cell culture of *T. monoccocum* L. with constructs containing the *GUS* gene driven by each of tested promoters (reporter constructs), and constructs containing each *MYB* TF gene driven by the constitutive polyubiquitin promoter (effector constructs) (Fig. 7A). The *GFP* gene cloned under the polyubiquitin promoter was used as a negative control to reveal basal levels of promoter activity in wheat cells.

As shown in Fig. 7B, TaMYB74 strongly activated all three tested promoters. A slightly milder activation of *TaATT1* and *TaKCS1* promoters was observed in TaMYB31 and TaMYB24, while TaMYB77 was unable to activate any promoter. The *TdSHN1* promoter was activated only by TaMYB74 (Fig. 7B).

### Identification of the functional MYB-responsive *cis*-elements in the *TdSHN1* promoter

**Fig. 8.**
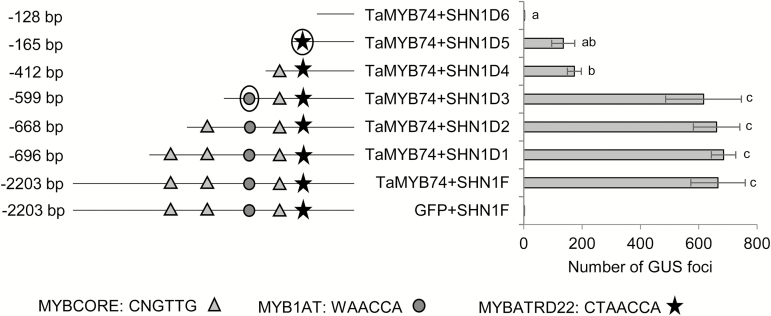
Identification of functional MYBR *cis*-elements in the *TdSHN1* promoter using a transient expression assay. The full-length *TdSHN1* promoter (F) and six 5'-deletions (D1–D6) were cloned upstream of the *GUS* reporter gene, and co-transformed by biolistic bombardment with either a negative control (pUbi–GFP) or pUbi–TaMYB74 constructs. Promoter deletions and existing MYBR *cis*-elements (MYBCORE, MYB1AT, and MYBATRD22) within 696bp upstream of the start codon are shown in the left panel. GUS expression quantifications are shown in the right side of the figure. One-way ANOVA with the Fisher’s least significant difference post-hoc test was conducted using GenStat. Error bars indicate the SE of three replicates. Functional *cis*-elements are circled.

The *TdSHN1* promoter was subjected to *cis*-element analysis using PLACE software (Higo *et al.*, 1999). Five potential MYBR elements were predicted within the 696bp of the cloned 2203bp long fragment upstream of the start codon of the *TdSHN1* gene, which will herein be referred to as the full-length promoter. To identify the functional MYBR *cis*-element(s), which is (are) specifically recognized by TaMYB74, a series of promoter deletions was generated at the 5' end of the full-length *TdSHN1* promoter. Deletions were generated in such a way that each of the five predicted MYBR *cis*-elements were removed one by one. As shown in Fig. 8, similar levels of *GUS* expression were initiated by the full-length promoter and by D1, D2, and D3 promoter deletions. However, the level of *GUS* expression driven by the D4 deletion decreased by ~70% compared with that driven by the D3 deletion. The 5'-AGGTGGTTATGC-3'/5'-GCATAACCACCT-3' sequence (the core sequence is underlined) designated here and below as the MYBR1 *cis*-element, predicted to be present on the promoter fragment between the D3 and D4 deletions, was the first (distal) functional MYBR *cis*-element. The second (proximal) functional MYBR *cis*-element, 5'-ATCTAACCACAT-3'/5'-ATGTGGTTAGAT-3' (the core sequence is underlined), designated as MYBR2, is situated between the D5 and D6 deletions. It was responsible for the remaining 30% of the *TdSHN1* promoter activity. The D6 deletion neither contained any MYBR elements nor provided any detectable activation of the *GUS* gene in wheat cells.

### Molecular model of TaMYB74 in complex with functional MYBR *cis*-elements identified in the *TdSHN1* promoter

**Fig. 9.**
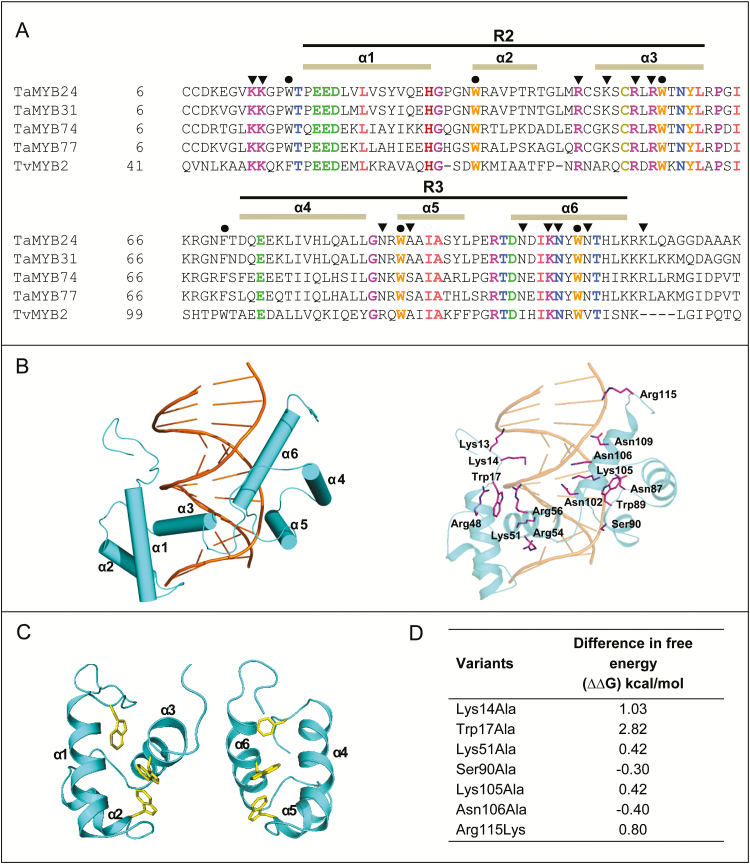
Protein sequence analyses and a molecular model of TaMYB74 in complex with the MYBR1 *cis*-element. (A) The protein alignments of the DNA-binding domains of wheat MYB and protozoan TvMYB2 proteins; the latter was used as a template for molecular modelling. Tandem imperfect amino acid repeats R2 and R3 are indicated by lines above the sequences. The conserved residues that form a hydrophobic core and the residues that interact with the DNA *cis*-element are denoted by filled circles and filled inverted triangles, respectively. (B) A cartoon of the TaMYB74 model (cyan) in complex with MYBR1 (orange) (left panel). Predicted residues interacting with DNA (distances between 2.8 Å and 3.6 Å) are shown in magenta sticks (right panel). (C) The orientations and positions of conserved tryptophan and phenylalanine residues, which form a hydrophobic core of TaMYB74. (D) Energy gains (kcal mol^–1^) upon mutation (into alanine or lysine) of Lys14, Trp17, Lys51, Ser90, Lys105, Asn106, and Arg115, involved in MYBR1 DNA binding, as determined by Fold-X (Schymkowitz *et al.*, 2005).

The TaMYB74 DNA-binding domain contains the conserved R2 and R3 repeats, and adopts a helix–turn–helix conformation with three regularly spaced tryptophan/phenylalanine residues, which form a hydrophobic core of the MYB domain (Fig. 9A, C). The binding domain consists of six α-helices: α1 (Gln19–His32), α2 (Trp37–Asp43), α3 (Gly50–Leu61), α4 (Phe72–Leu85), α5 (Trp89–Arg95), and α6 (Asp101–Arg113). The binding domain binds the MYBR1 DNA element through the α3 helix (R2 motif) and the α6 helix (R3 motif) in the major groove of DNA and makes contacts by forming hydrogen bonds between charged (Lys14, Lys51, and Lys105) and polar residues (Asn102, Asn106, and Asn109) with nucleo-bases (Fig. 9B; Supplementary Table S2). In addition, the hydrogen bonds are mediated by Lys13 and Trp17 at the N-terminus, Arg48, Arg54, and Arg56 of the α3 helix, Asn87, Trp89, and Ser90 of the α5 helix, and Arg115 at the C-terminus, to the sugar-phosphate DNA backbone, to increase the overall stability of the complex (Supplementary Table S2). Hydrogen bond distances between residues and nucleo-bases or sugar-phosphate backbones are between 2.8 Å and 3.6 Å.

TaMYB74 also binds to MYBR2. MYBR2 is similar to MYBR1 and contains complementary nucleotides to the core sequence of MYBR1. To analyse which side of the DNA element is bound to the protein, its conformational stability was calculated using Fold-X force-field (Schymkowitz *et al.*, 2005). The free energies (∆*G*) of TaMYB74 with ‘TGGTTA’ and ‘TAACCA’ were 67.5 kcal mol^–1^ and 74.2 kcal mol^–1^, respectively, meaning that TaMYB74 is more stable when forming a complex at the ‘TGGTTA’ site. We predicted why TaMYB74 binds less strongly to MYBR2 than to MYBR1. The differences of DNA sequences in both *cis*-elements affect DNA conformation and orientation. In Supplementary Table S2 we show that nine residues are involved in forming hydrogen bonds to the core sequence of the MYBR1 sense strand. Although MYBR2 also contains the core sequence, the neighbouring nucleotides may affect the conformation of core nucleo bases T_4_, T_7_, and T_8_, and result in a loss of binding to Asn109, Lys14, and Lys51.

The crystal structure of TvMYB2 in complex with MRE-1–12 indicates that the protein interacts with MRE-1–20 DNA via Lys49 (contact with T_3_' and T_5_), Arg84 (contact with G_5_'), Lys138 (contact with G_3_), and Asn139 (contact with A_2_'), which are responsible for sequence-specific recognition (Jiang *et al.*, 2011). TvMYB2 also interacts with a DNA phosphate backbone via Lys48, Lys49, Gln50, Phe52, Gln85, Arg87, Arg89, Tyr93, Arg120, Trp122, Ala123, and Asn146. Notably, binding affinity is reduced with single residue substitutions at Lys51, Phe52, and Arg87 with alanine. The Arg84Ala change is particularly effective, whereby it completely removes binding to MRE-1–20 (Jiang *et al.*, 2011).

Based on the TvMYB2 crystal structure, we predicted critical residues in TaMYB74 by calculating energy gains (kcal mol^–1^) upon mutation of specific DNA-binding residues to alanine, using Fold-X force-field (Schymkowitz *et al.*, 2005) (Fig. 9D). The binding affinity of TaMYB74 for the DNA *cis*-element may not change for Asn106Ala, and substitutions of Lys51Ala and Lys105Ala that correspond to Arg84 and Lys139 in TvMYB2, respectively, which are predicted to have little destabilizing effect (Fig. 9D). The most destabilizing substitutions in TaMYB74 are those of Lys14Ala and Trp17Ala, which correspond to Lys49 and Phe52 in TvMYB2, respectively. As for energy contributions, it has been estimated that a loss of 1 kcal mol^–1^ corresponds to approximately one hydrogen bond (Fersht, 1987). There are 15 residues involved in DNA binding in TaMYB74 (Fig. 9B). Two of these, Ser90 and Arg115, are different in TaMYB24 and TaMYB31 (alanine and lysine, respectively; Fig. 9A). Based on calculated free energies, Ser90Ala may not affect protein–DNA interaction, while Arg115Lys is projected to destabilize it slightly (Fig. 9D).

## Discussion

It has been established that the primary functions of cuticle and, particularly, of the cuticular waxes is in the protection against excessive solar irradiation and conservation of internal plant water (Yeats and Rose, 2013). Accumulation of epicuticular waxes on plant surfaces often results in a bluish-white coloration termed glaucousness. As a crop trait, glaucousness increases light reflectance and reduces leaf temperature and transpiration, thereby enhancing leaf survival under water deficit and improving water use efficiency (WUE) (Richards *et al.*, 1986; Febrero *et al.*, 1998).

The genetic analysis of variation in ﬂag leaf glaucousness in Australian wheat cultivars has revealed numerous loci inﬂuencing this trait (Bennett *et al.*, 2012*a*), indicating complex genetic and metabolic control. However, the extent of deployment of this control within locally adapted germplasm is unknown.

In this work, data on contrasting Australian wheat cultivars, RAC875 (glaucous, drought tolerant) and Kukri (non-glaucous, drought sensitive), previously characterized in terms of stress physiology, genetics, and metabolomics (Izanloo *et al.*, 2008; Bennett *et al.*, 2012*a*, *b*; Bowne *et al.*, 2012), were used with the aim to: (i) uncover the biochemical background of differences in glaucousness; (ii) identify genes coding for MYB TFs that potentially may be involved in the regulation of cuticular wax biosynthesis pathways under drought; (iii) functionally characterize selected MYB TFs in their ability to activate promoters of genes involved in cuticle biosynthesis; (iv) develop a 3D molecular model of TF–DNA binding interactions, and (v) establish the link between the activity of characterized MYB TFs and cuticular wax composition.

As a first step, it was demonstrated that the difference in leaf glaucousness of RAC875 and Kukri (leaf waxiness indices are 4 and 5 for RAC875, and 1 and 1.5 for Kukri, grown under well-watered conditions or mild drought conditions, respectively) (Izanloo *et al.*, 2008) is probably caused by the presence of significant amounts of β-diketones in the wax of RAC875 (Fig. 1). A number of studies suggested that β-diketones might be responsible for the glaucous appearance of wheat and barley (Adamski *et al.*, 2013; Zhang *et al.*, 2013), and our study strongly supports this assumption. Moreover, the recently identified gene clusters responsible for accumulation of β-diketones in barley (Schneider *et al.*, 2016) and wheat (Hen-Avivi *et al.*, 2016) were localized in the *W1* locus on chromosome 2BS, which was previously shown to be the determinant for glaucousness. Using a double-haploid population of RAC875 and Kukri, Bennett *et al.* (2012*a*) identified the *QW.aww-2B-1* quantitative trait locus, at a position similar to that of *W1* on chromosome 2B, that affected glaucousness. Combined together, these data open up a new opportunity for further detailed genetic analysis of glaucousness and biosynthesis of β-diketones in Australian wheats.

During growth under limited watering (mild drought), the amounts of waxes increased in both RAC875 and Kukri without changes in the shapes of wax crystals (Fig. 1). While β-diketone content did not increase in response to drought in either RAC875 or Kukri, both cultivars under drought accumulated elevated amounts of primary alcohols and alkanes; this observation correlates well with findings of other plant species in response to a limited water supply (Bernard and Joubès, 2013; Yeats and Rose, 2013). The accumulation of very long chain alcohols (C-28) and alkanes (C-29, C-31) suggested the activation of enzymes involved in fatty acid elongation (FAE) pathways (von Wettstein-Knowles, 2012).

As a second step, we characterized six wheat *MYB* genes, which were cloned from RAC875 based on sequence homology of their products to known cuticle biosynthesis-related MYB TFs from Arabidopsis. To our knowledge, no homologues of these five Arabidopsis MYB TFs from cereals have been yet characterized, with the exception of ZmMYB94 from maize (La Rocca *et al.*, 2015). Expression of the cloned genes was analysed in RAC875 and Kukri under two types of dehydration stresses, as described below. The impact of regulatory genes such as TF genes during stress is often rapid and transient: after some time, the levels of transcripts return to initial levels, even if the stress factor is persisting. Consequently, if stress develops slowly, as usually occurs in the case of drought in the field, changes in expression of TF genes with a short transient response are often difficult to measure. For these reasons,we used two regimes of dehydration: (i) rapid dehydration of detached leaves at ambient temperature; and (ii) slowly developing (within several days or weeks) and repeatable (cyclic) drought of plants growing in soil (Supplementary Fig. S1). The aim of the first experiment was to detect rapid and transient changes in expression of *MYB* genes. The aim of the second experiment was to compare differences in basal levels of gene expression, and to detect long-lasting and late changes in expression levels under the conditions of two successive cycles of drought.

We found that two TF genes, *TaMYB31* and *TaMYB74*, were up-regulated and two other genes, *TaMYB24* and *TaMYB77*, were down-regulated by both rapid dehydration and slowly developing drought. The remaining two genes, *TaMYB16* and *TaMYB78*, showed no expression in leaves either under well-watered conditions or under drought, and therefore we did not study these further. In all cases, cultivar-specific differences in gene expression were found under rapid dehydration. For example, both *TaMYB31* and *TaMYB74* reached the highest expression levels earlier during dehydration in Kukri than in RAC875, possibly reflecting the sensitivity of these TFs to a common threshold level of dehydration. Detached leaves of Kukri reached the threshold dehydration state earlier than leaves of RAC875. Up-regulation of *TaMYB31* in drought-tolerant RAC875 was stronger than in Kukri, suggesting that higher levels of expression of *TaMYB31* might be one of the reasons for the higher drought tolerance of RAC875. In contrast, a difference in maximal induction levels between the two wheat cultivars was not observed for the *TaMYB74* gene, suggesting a universal requirement for its product under dehydration. The transcript numbers of *TaMYB24* slightly decreased in RAC875 and then rapidly returned to initial levels. This was different from the behaviour of this gene in Kukri, perhaps suggesting that the dehydration response of *TaMYB24* expression is critical for drought tolerance.

The comparison of *TaMYB31* and *TaMYB74* expression levels in plants growing under slowly developing drought revealed significant differences in the time of gene induction during progression of stress, and hence possible dissimilarities in functions of these two genes. *TaMYB31* is an early stress-responsive gene with transient expression, which starts to normalize when a plant is still under strong stress. In contrast, the expression levels of *TaMYB74* were only moderately influenced by mild stress, but were strongly elevated when dehydration became critical (at wilting). In addition, *TaMYB74* did not react on the second cycle of drought in RAC875, which might indicate that the product of this gene is more stable in the drought-tolerant cultivar. These differences between *TaMYB31* and *TaMYB74* expression were less obvious in the rapid leaf dehydration experiment. Up-regulation of *TaMYB74* and *TaMYB31* by both rapid dehydration and cyclic drought were in accordance with the data obtained for their Arabidopsis counterparts, *AtMYB41* and *AtMYB96*. Both Arabidopsis TFs have been reported to be up-regulated by environmental stresses and play multiple roles in response to drought and osmotic stress (Lippold *et al.*, 2009; Seo *et al.*, 2009; Seo and Park, 2010). Besides regulating the amount and quality of cuticle, these TFs might confer drought tolerance through different pathways, such as through the regulation of stomatal development (Yang *et al.*, 2011).

The analysis of gene expression levels in different wheat tissues revealed similarities in the expression patterns of *TaMYB24* and *TaMYB31* (Fig. 6). The only notable difference was observed in the levels of expression in bracts. A similar tissue distribution of *TaMYB24* and *TaMYB31* may reflect their high level of homology (49.5% sequence identity at the protein level); both proteins represent wheat counterparts of MYB96 from Arabidopsis. However, changes in the expression levels of these two genes occurred in opposite directions under stress. Possible explanations for such a different reaction to stress are: (i) these genes have different tissue- or cell layer-specific patterns of expression, which cannot be detected by Q-PCR, and thus would provide different patterns of tissue- or cell layer-specific regulation of the same target genes under stress; or (ii) small variations in protein sequences of DNA-binding domains of *TaMYB24* and *TaMYB31* exist, which are sufficient to provide different DNA binding specificity and hence activation of different groups of target genes.

In the absence of stress, *TaMYB74* was mostly expressed in roots. This finding correlates with recent data about the involvement of its Arabidopsis counterpart, *MYB41*, in the synthesis and deposition of suberin, a polymer which is similar to cutin and is localized mostly in root endodermis and peridermis, and in the seed coat of Arabidopsis (Vishwanath *et al.*, 2013; Kosma *et al.*, 2014). The relatively high levels of *TaMYB74* expression in other tissues, including leaves, as well as strong induction of this gene by drought may suggest its involvement in the regulation of a number of other biochemical and physiological processes in wheat. Similarly to earlier reports for Arabidopsis *MYB16* (Oshima and Mitsuda, 2013), the highest level of expression of *TaMYB77* was found in vegetative tissues. However, expression of this gene in immature inflorescence and developing grain was also elevated compared with other tissues, suggesting the possible involvement of these genes in wheat organ development.

A transient expression assay in wheat suspension cells was used to confirm the participation of drought-affected wheat MYB TFs in transcriptional activation of cuticle-related genes (Fig. 7). For this purpose, the wheat homologues of the Arabidopsis *ATT1* gene, encoding an enzyme from the cutin biosynthetic pathway, the *KCS1* gene, encoding an enzyme from the wax biosynthetic pathway, and *WIN1*/*SHN1*, encoding the regulator of wax biosynthesis (Yeats and Rose, 2013; Borisjuk *et al.*, 2014), were identified, and promoters of wheat genes were designated as *TaATT1*, *TaKCS1*, and *TdSHN1*, respectively. These were selected for the assay because it was earlier reported that overexpression of *MYB41* in Arabidopsis activated *ATT1* and *WIN1*/*SHN1* genes, while the *KCS1* gene was activated by overexpression of *MYB96* in both a transcription activation assay and transgenic Arabidopsis, and overexpression of *MYB16* in transgenic Arabidopsis led to activation of *SHN1* and *KCS1* (Borisjuk *et al.*, 2014). In our assay, three of the four tested wheat TFs, TaMYB74, TaMYB31, and TaMYB24, activated either two or three cloned wheat promoters, and therefore can be considered as true cuticle biosynthesis-related genes in wheat. Surprisingly, these three wheat MYB TFs demonstrated less selectivity in target gene activation than their corresponding Arabidopsis homologues. Each of the wheat MYBs could activate both *TaATT1* and *TaKCS1* promoters, although with variable efficiency. On the other hand, no activation of the *TdSHN1* gene was seen with TaMYB77, although *SHN1* is reported to be the target gene of the Arabidopsis homologue AtMYB16. An absence of activation by TaMYB31 and TaMYB24 can be explained by the absence of the TAACTA/G type of MYBR *cis*-elements in the *TdSHN1* promoter, which are specifically recognized by Arabidopsis MYB96 and might also be specific for the wheat homologues (Seo *et al.*, 2011).

A transient expression assay has been used in this work in combination with molecular modelling for the identification of possible differences in the recognition of MYBR *cis*-element(s) in the *TdSHN1* promoter. Mapping of the promoter, using a series of promoter deletions, revealed two similar MYBR elements which were specifically recognized only by TaMYB74 (Fig. 8). The distal element, designated MYBR1, was responsible for ~70% of promoter activation by TaMYB74, while the proximal element (MYBR2) accounted for the remaining 30%.

The molecular model of TaMYB74 in complex with functional *cis*-elements from the *TdSHN1* promoter suggested that small, but central differences in nucleotides that are adjacent to the same core sequence TGGTTA, may explain differences in the apparent efficiency of promoter activation through MYBR1 and MYBR2 *cis*-elements. However, no significant differences were found between the DNA-binding domains of TaMYB74 and TaMYB24, TaMYB31 and TaMYB77, that would explain the selectivity of recognition of the *TdSHN1* promoter (Fig. 9). A mechanistic explanation for why the *TdSHN1* promoter was activated only by TaMYB74 remains to be determined.

**Scheme 1.**
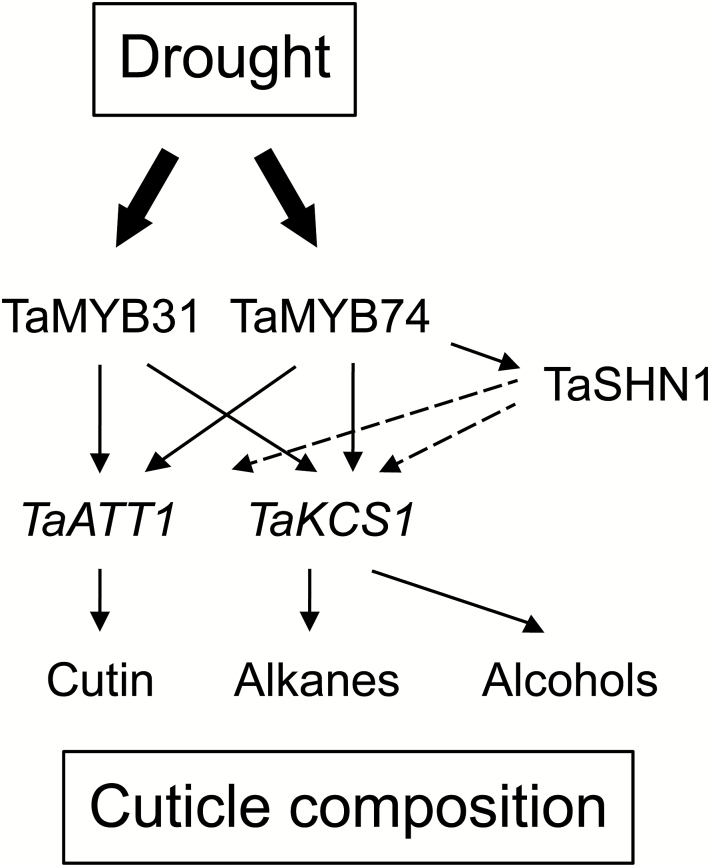
The proposed roles of TaMYB31 and TaMYB74 in the regulation of cuticle biosynthesis under drought. The dashed lines reflect the roles of TaSHN1 in regulating TaATT1 and TaKCS1 genes, and consequently the biosynthesis of cuticular wax components, based on our own and other data.

Scheme 1 summarizes our findings on activation of cuticle biosynthetic pathways in wheat by the TFs TaMYB31 and TaMYB74, homologues of the well-characterized cuticle biosynthesis regulators AtMYB96 (Seo *et al.*, 2011) and AtMYB41 (Cominelli *et al.*, 2008). Expression of both wheat MYB TFs was up-regulated by drought (Fig. 5), and both TFs activated *ATT1* and *KCS1* genes through direct binding to their promoters (Fig. 7). Activation of the *TaKCS1* gene that encodes a key enzyme in the FAE pathway (Bernard and Joubès, 2013) may explain the increased accumulation of very long chain alkanes and primary alcohols in response to drought. In a cyclic drought experiment (Fig. 5), the expression levels of *TaMYB31* peaked much earlier (day 5) compared with those of TaMYB74 (day 14); these observations are consistent with its more specialized role in the regulation of a cutin biosynthesis, which under drought starts earlier than the biosynthesis of cuticular waxes (Bernard and Joubès, 2013). The *TaMYB74* gene in turn possibly plays a more general role in cuticle biosynthesis, which is in agreement with specific activation by TaMYB74 of the *SHN1* gene, and hence with a position of this TF upstream of *TaSHN1* in the hierarchy of cuticle biosynthesis regulators.

In summary, we revealed that β-diketones are the main compositional determinants in the two elite Australian wheat cultivars RAC875 and Kukri, underlying the glaucous and non-glaucous phenotypes, respectively. The concentration of β-diketones remained unchanged during growth of both cultivars under limited watering, while the content of other wax components, alkanes and primary alcohols, increased. These findings suggest that a combination of β-diketones and stress-stimulated accumulation of other cuticle compounds may make RAC875 more resistant to a water loss under drought. We demonstrated drought-inducible expression of four isolated wheat *MYB* genes. Products of three genes (*TaMYB74, TaMYB31*, and *TaMYB24*) operated as the activators of cuticle biosynthetic genes in wheat cells. Moreover, two functional MYB-responsive elements localized in the promoter region of the *SHN1* gene were specifically recognized by TaMYB74, but not by other MYB TFs. We revealed the protein structural determinants underlying the binding specificity of two identified functional DNA *cis*-elements by TaMYB74, one of the investigated wheat TFs. We have integrated our data with other observations, and propose a scheme that links drought, the investigated TFs, downstream cuticle-related biosynthetic genes, and cuticle wax components. Our results extend the knowledge on cuticle biosynthesis regulation in grasses and can potentially be used for engineering of cereal crops with enhanced tolerance and performance under drought.

## Supplementary data

Supplementary data are available at *JXB* online.

Figure S1. Schematic diagram of the cyclic drought experiment (modified from Harris *et al.*, 2016).

Figure S2. Amounts of wax components in RAC875 (RAC) and Kukri (KUK) grown under well-watered (WW) and mild drought (DR) conditions.

Figure S3. A schematic representation showing the gene structure of the six wheat MYB TFs investigated in this study.

Table S1. List of primers used in this study.

Table S2. Amino acid residues of TaMYB74 forming hydrogen bonds with 12bp DNA *cis*-elements of MYBR1 (5'-AGGTGGTTATGC-3'/5'-GCATAACCACCT-3') and MYBR2 (5'-ATCTAACCACAT-3'/5'-ATGTGGTTAGAT-3'). The core binding sequence in *cis*-elements is underlined.

Supplementary Data
