# Supplementary material for: Identification and characterization of wheat drought-responsive MYB transcription factors involved in the regulation of cuticle biosynthesis
Source: J Exp Bot. 2016 Aug 3;67(18):5363–80. doi: 10.1093/jxb/erw298 (PMC5049387; doi:10.1093/jxb/erw298)
Supplement: Supplementary Data [file supp_67_18_5363__index.html]

Identification and characterization of wheat drought-responsive MYB transcription factors involved in the regulation of cuticle biosynthesis — Identification and characterization of wheat drought-responsive MYB transcription factors involved in the regulation of cuticle biosynthesis — Supplementary Data 

# Identification and characterization of wheat drought-responsive MYB transcription factors involved in the regulation of cuticle biosynthesis

## Supplementary Data

Data files

- Supplementary\_Information\_Supplementary\_figures\_S1\_S3\_Tables\_S1\_S2.pdf - Supplementary Data
