# Supplementary material for: Identification and characterization of wheat drought-responsive MYB transcription factors involved in the regulation of cuticle biosynthesis
Source: J Exp Bot. 2016 Aug 3;67(18):5363–80. doi: 10.1093/jxb/erw298 (PMC5049387; doi:10.1093/jxb/erw298)
Supplement: Supplementary Data [file supp_erw298_Supplementary_Information_Supplementary_figures_S1_S3_Tables_S1_S2.pdf]

Identification and characterisation of wheat drought-responsive MYB transcription factors involved in the regulation of cuticle biosynthesis

Huihui Bi[1], Sukanya Luang[1], Yuan Li[1], Natalia Bazanova[1], Sarah Morran[1], Zhihong Song[2], M. Ann Perera[2], Maria Hrmova[1]*, Nikolai Borisjuk[1], Sergiy Lopato[1]

[1]*Australian Centre for Plant Functional Genomics, School of Agriculture, Food and Wine, University of Adelaide, Glen Osmond, South Australia 5064, Australia*
[2]*W.M.Keck Metabolomics Research Laboratory, Iowa State University, Ames, IA 50011, USA*

Supplementary Introduction

Protection against extreme ultraviolet (UV) radiation, prevention of dehydration, tolerance to high salinity and cold stress, as well as resistance to pest and pathogens are reported as the major functions of the cuticle (Amid *et al.*, 2012; Bourdenx *et al.*, 2011; Goodwin and Jenks, 2005; Kosma *et al.*, 2009; Kosma *et al.*, 2010; Lee *et al.*, 2014; Panikashvili *et al.*, 2007; Shepherd and Wynne Griffiths, 2006; Uppalapati *et al.*, 2012; Wang *et al.*, 2014; Wang *et al.*, 2012; Zhang *et al.*, 2007). It is well documented that drought can induce increased wax depositions on the leaf and stem surfaces of many plant species, such as *Arabidopsis*, cotton, soybean, rice, sesame, rose, peanut, ficus and tree tobacco (*Nicotiana glauca*) (Bondada *et al.*, 1996; Cameron *et al.*, 2006; Jenks *et al.*, 2001; Kim *et al.*, 2007a; Kim *et al.*, 2007b; Kim, 2008; Kosma *et al.*, 2009; Samdur *et al.*, 2003; Zhu and Xiong, 2013). A naturally occurring mutant of wild barley (*Hordeum spontaneum*), *eibi1*, which has a very thin cuticle layer, is sensitive to drought (Chen *et al.*, 2011). Similarly, a rice EMS mutant *wsl2* has approximately 80% less total wax content and is also more sensitive to drought than wild type rice (Mao *et al.*, 2012).

Supplementary Results

Gene cloning and the phylogenetic relationships of MYB TFs

Three of the cloned wheat genes, a homologue of *AtMYB106* and two homologues of

AtMYB96, encode either the same or highly similar protein sequences to TaMYB16 (GenBank accession AEV91158.1, 100% identity), TaMYB24 (GenBank accession AEV91147.1, 99% identity) and TaMYB31 (GenBank accession AEV91154.1, 98% identity), respectively (Zhang *et al.*, 2012). These very high levels of identity with protein sequences reported by Zhang *et al.* (2012) are suggestive of homeologues or cultivar- and/or allele-specific origins. These three genes will be referred to as *TaMYB16*, *TaMYB24* and *TaMYB31*. The other three cloned wheat genes that are homologous to *AtMYB41*, *AtMYB16* and *SlMYB12* are novel and, therefore, these three wheat gene sequences were named *TaMYB74*, *TaMYB77* and *TaMYB78*, respectively.

Selection of MYB genes that are regulated by water deficit

Before dehydration *TaMYB24* had about 1.5-fold higher basal level of expression in Kukri than in RAC875. However, after two hours of dehydration the basal level of expression dropped 3.5-fold in leaves of Kukri and 1.5-fold in RAC875. In the drought-sensitive cultivar Kukri, the number of transcripts continued to decrease with dehydration. By contrast, in the leaves of the drought-tolerant cultivar RAC875, the expression levels of *TaMYB24* returned to initial levels after seven hours of dehydration.

Gene expression levels of *TaMYB31* in the absence of drought in the flag leaves of both Kukri and RAC875 cultivars were equally low. After the first two hours of dehydration, the expression of *TaMYB31* increased about two-fold in both wheat cultivars. Subsequently, expression returned to initial levels in Kukri, but kept increasing in RAC875, where after seven hours of leaf dehydration, expression was around six-fold higher than initial levels (Fig. 4).

The expression levels of *TaMYB74* gene during dehydration were different in wheat cultivars with contrasting drought tolerance. The basal levels of *TaMYB74* expression were low in both cultivars. In Kukri, the number of transcripts increased rapidly to about four-fold after two hours of dehydration, compared to the initial number of transcripts, and remained at the same level during next five hours of dehydration. In RAC875, however, the number of transcripts increased gradually with dehydration and reached a similar level to Kukri of a five-fold increase after seven hours of leaf dehydration (Fig. 4).

The basal expression levels and responses to dehydration of *TaMYB77* were similar in Kukri and RAC875. After two hours of dehydration the relatively low basal level of *TaMYB77* expression decreased three-fold in both cultivars and the same number of *TaMYB77* transcripts prevailed until the end of the experiment (Fig. 4).

The induction of *TaMYB24*, *TaMYB31*, *TaMYB74* and *TaMYB77* expression by drought was investigated in the flag leaves of wheat cultivars Kukri and RAC875 during three consecutive cycles of drought (Fig. 5). Water status during the experiment and the time points of leaf sampling are shown in Supplementary Fig. S1. The basal levels of *TaMYB24* expression were much higher than those of the other three *MYB* genes. The overall levels of expression of *TaMYB24* under cyclic drought were about two-fold and 1.5-fold lower than those under sufficient watering of Kukri and RAC875, respectively.

In contrast, the expression levels of *TaMYB31* gene under mild drought conditions (5 days after last watering) were 2.5-fold higher than in well-watered Kukri plants compared to a 1.5-fold increase in transcripts in RAC875 plants. The numbers of *TaMYB31* transcripts decreased with time under both well-watered and drought conditions, suggesting a developmental dependency of *TaMYB31* expression.

In Kukri, the transcript numbers of *TaMYB74* were dramatically increased only at fourteenth day of the first cycle of drought, when drought was strong and wheat plants started to wilt; the similar increase in transcripts was repeated at the end of a second cycle of drought (day 23). Similarly, the significant increase of *TaMYB74* transcripts was observed in the RAC875 flag leaves at fourteenth day; however there was no response of this gene during the second cycle of drought.

The number of *TaMYB77* transcripts in Kukri started to decrease at the ninth day of the first drought cycle and continued until the fourteenth day. The numbers of transcripts were significantly decreased at the end of the second cycle of drought and returned to normal levels after re-watering. In RAC875, the number of transcripts under drought were overall slightly reduced compared to those under well-watered conditions, but were not dependent on drought strength.

Supplementary References

Amid A, Lytovchenko A, Fernie AR, Warren G, Thorlby GJ. 2012. The *sensitive to freezing3* mutation of *Arabidopsis thaliana* is a cold-sensitive allele of homomeric acetyl-CoA carboxylase that results in cold-induced cuticle deficiencies. Journal of Experimental Botany **63**, 5289-5299.

Bondada BR, Oosterhuis DM, Murphy JB, Kim KS. 1996. Effect of water stress on the epicuticular wax composition and ultrastructure of cotton (*Gossypium hirsutum* L.) leaf, bract, and boll. Environmental and Experimental Botany **36**, 61-69.

Bourdenx B, Bernard A, Domergue F, Pascal S, Léger A, Roby D, Pervent M, Vile D, Haslam RP, Napier JA, Lessire R, Joubès J. 2011. Overexpression of Arabidopsis ECERIFERUM1 promotes wax very-long-chain alkane biosynthesis and influences plant response to biotic and abiotic stresses. Plant Physiology **156**, 29-45.

Bowne JB, Erwin TA, Juttner J, Schnurbusch T, Langridge P, Bacic A, Roessner U. 2012. Drought responses of leaf tissues from wheat cultivars of differing drought tolerance at the metabolite level. Molecular Plant **5**, 418-429.

Cameron KD, Teece MA, Smart LB. 2006. Increased accumulation of cuticular wax and expression of lipid transfer protein in response to periodic drying events in leaves of tree tobacco. Plant Physiology **140**, 176-183.

Chen G, Komatsuda T, Ma JF, Nawrath C, Pourkheirandish M, Tagiri A, Hu Y-G, Sameri M, Li X, Zhao X. 2011. An ATP-binding cassette subfamily G full transporter is essential for the retention of leaf water in both wild barley and rice. Proceedings of the National Academy of Sciences of the United States of America **108**, 12354-12359.

Goodwin S, Jenks M. 2005. The plant cuticle involvement in drought tolerance. In: Jenks M, Hasegawa P, eds. *Plant Abiotic Stress*. Oxford: Blackwell Scientific Publishers, 14-36.

Jenks MA, Andersen L, Teusink RS, Williams MH. 2001. Leaf cuticular waxes of potted rose cultivars as affected by plant development, drought and paclobutrazol treatments. Physiologia Plantarum **112**, 62-70.

Kim KS, Park SH, Jenks MA. 2007a. Changes in leaf cuticular waxes of sesame (*Sesamum indicum* L.) plants exposed to water deficit. Journal of Plant Physiology **164**, 1134-1143.

Kim KS, Park SH, Kim DK, Jenks MA. 2007b. Influence of water deficit on leaf cuticular waxes of soybean (*Glycine max* [L.] Merr.). International Journal of Plant Sciences **168**, 307-316.

Kim KW. 2008. Visualization of micromorphology of leaf epicuticular waxes of the rubber tree *Ficus elastica* by electron microscopy. Micron **39**, 976-984.

Kosma DK, Bourdenx B, Bernard A, Parsons EP, Lü S, Joubès J, Jenks MA. 2009. The impact of water deficiency on leaf cuticle lipids of *Arabidopsis*. Plant Physiology **151**, 1918-1929.

Kosma DK, Nemacheck JA, Jenks MA, Williams CE. 2010. Changes in properties of wheat leaf cuticle during interactions with Hessian fly. The Plant Journal **63**, 31-43.

Lee SB, Kim H, Kim RJ, Suh MC. 2014. Overexpression of *Arabidopsis MYB96* confers drought resistance in *Camelina sativa* via cuticular wax accumulation. Plant Cell Reports **33**, 1535-1546.

Mao B, Cheng Z, Lei C, *et al.* 2012. Wax crystal-sparse leaf2, a rice homologue of WAX2/GL1, is involved in synthesis of leaf cuticular wax. Planta **235**, 39-52.

Panikashvili D, Savaldi-Goldstein S, Mandel T, Yifhar T, Franke RB, Höfer R, Schreiber L, Chory J, Aharoni A. 2007. The *Arabidopsis* DESPERADO/AtWBC11 transporter is required for cutin and wax secretion. Plant Physiology **145**, 1345-1360.

Samdur M, Manivel P, Jain V, Chikani B, Gor H, Desai S, Misra J. 2003. Genotypic differences and water-deficit induced enhancement in epicuticular wax load in peanut. Crop Science **43**, 1294-1299.

Shepherd T, Wynne Griffiths D. 2006. The effects of stress on plant cuticular waxes. New Phytologist **171**, 469-499.

Uppalapati SR, Ishiga Y, Doraiswamy V, Bedair M, Mittal S, Chen J, Nakashima J, Tang Y, Tadege M, Ratet P. 2012. Loss of abaxial leaf epicuticular wax in *Medicago truncatula irg1*/*palm1* mutants results in reduced spore differentiation of anthracnose and nonhost rust pathogens. The Plant Cell **24**, 353-370.

Wang J, Li W, Wang W. 2014. Fine mapping and metabolic and physiological characterization of the glume glaucousness inhibitor locus *Iw3* derived from wild wheat. Theoretical and Applied Genetics **127**, 831-841.

Wang Y, Wan L, Zhang L, Zhang Z, Zhang H, Quan R, Zhou S, Huang R. 2012. An ethylene response factor OsWR1 responsive to drought stress transcriptionally activates wax synthesis related genes and increases wax production in rice. Plant Molecular Biology **78**, 275-288.

Zhang JY, Broeckling CD, Sumner LW, Wang ZY. 2007. Heterologous expression of two *Medicago truncatula* putative ERF transcription factor genes, *WXP1* and *WXP2*, in *Arabidopsis* led to increased leaf wax accumulation and improved drought tolerance, but differential response in freezing tolerance. Plant Molecular Biology **64**, 265-278.

Zhang L, Zhao G, Jia J, Liu X, Kong X. 2012. Molecular characterization of 60 isolated wheat MYB genes and analysis of their expression during abiotic stress. Journal of Experimental Botany **63**, 203-214.

Zhu X, Xiong L. 2013. Putative megaenzyme DWA1 plays essential roles in drought resistance by regulating stress-induced wax deposition in rice. Proceedings of the National Academy of Sciences of the United States of America **110**, 17790-17795.

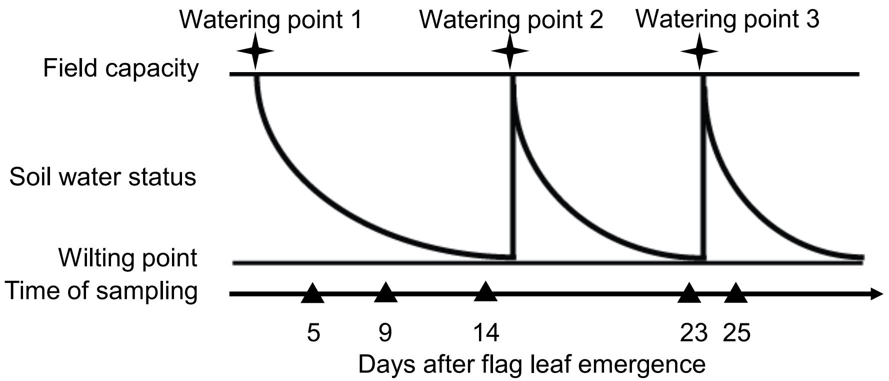

Supplementary Fig. S1

Schematic diagram of the cyclic drought experiment (modified from Bowne et al., 2012). Plants were watered at three time points as indicated by stars. Soil water content gradually decreased after watering until wilting point, at which water was re-applied. Leaf samples for RNA extraction were collected at five time points (5, 9, 14, 23 and 25 days after initial withholding of water), as indicated by triangles.

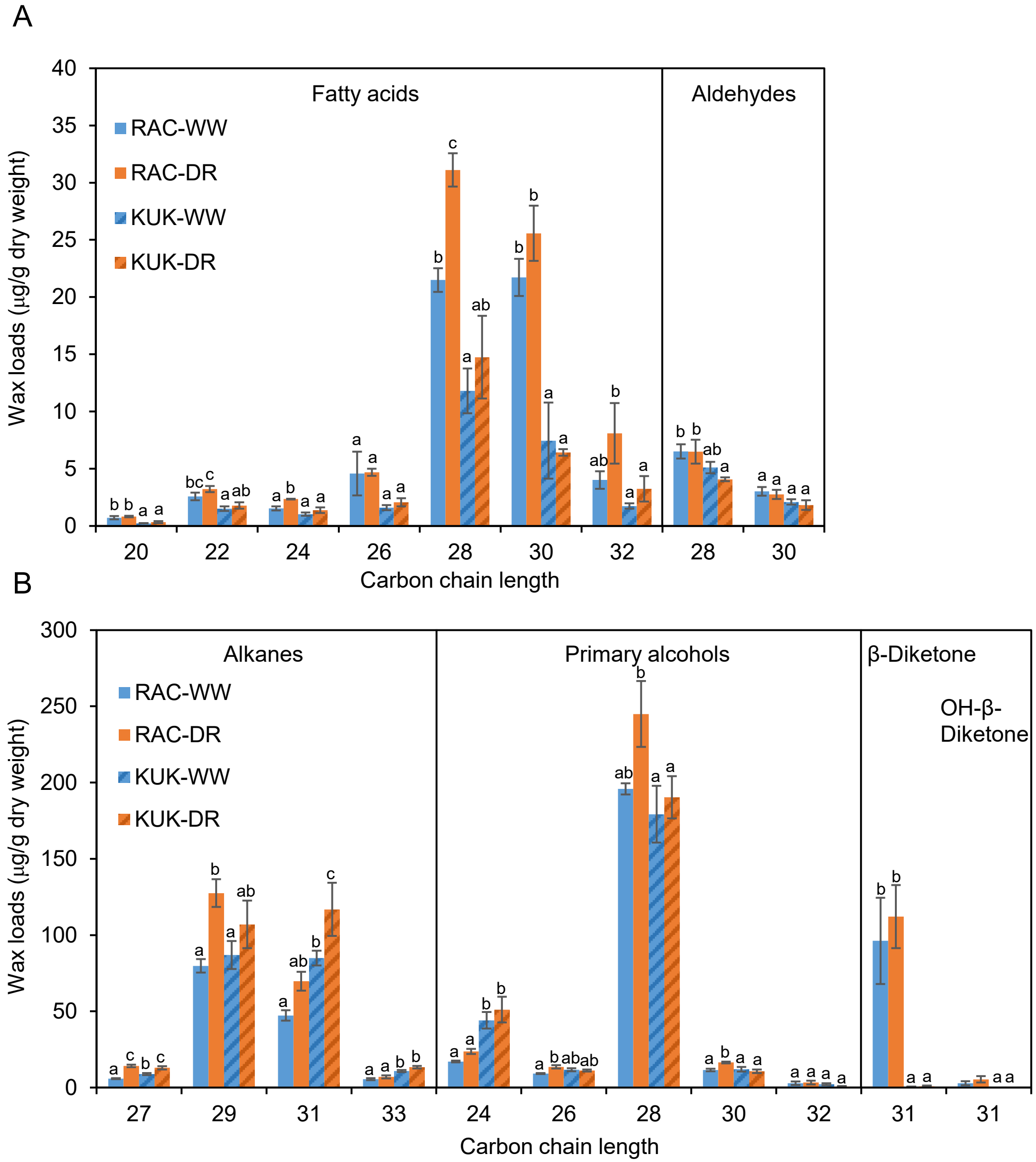

Supplementary Fig. S2

Amounts of wax components in RAC875 (RAC) and Kukri (KUK) grown under well-watered (WW) and mild drought (DR) conditions. A, Amounts of fatty acids and aldehydes. B, Amounts of alkanes, primary alcohols and β-diketones. Low amounts of C23, C25 alkanes, C20, C22, C34 primary alcohols and resorcinols in both cultivars, grown under the two conditions, are not shown but were included in the calculations of total wax loads (Fig. 1F). Means and standard errors were calculated from three replicates. Two-way ANOVA with the Fisher's Least Significant Difference *post hoc* test was conducted using GenStat. Small letters on the top of error bars indicate differences that are not significant at the 5% level.

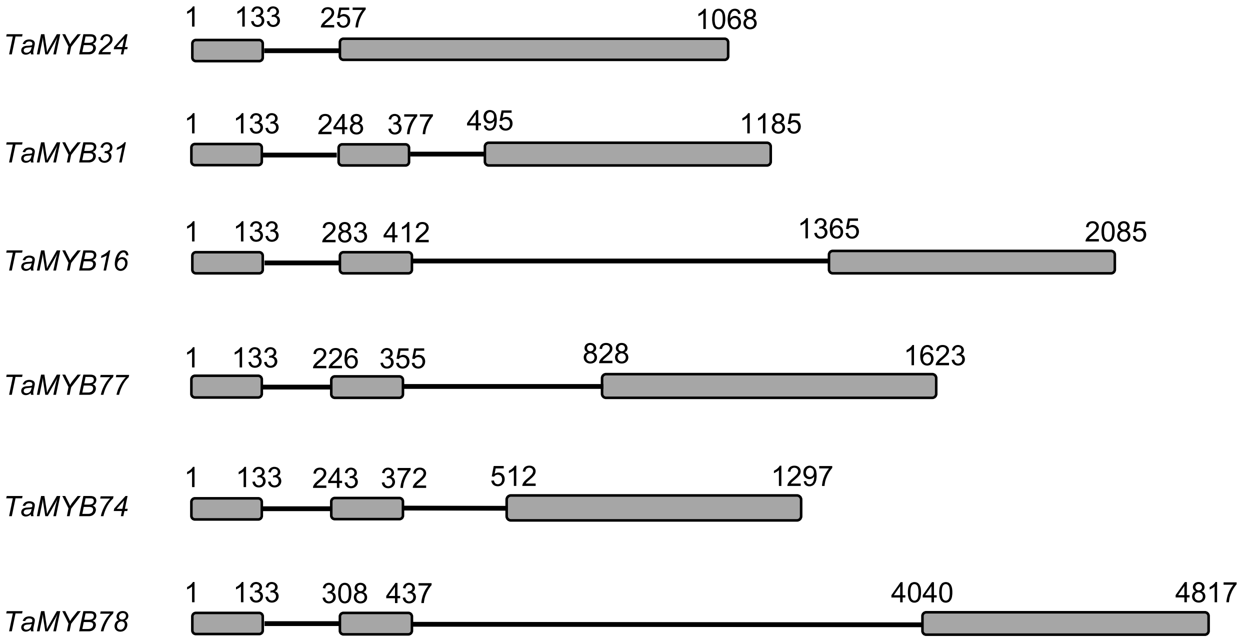

Supplementary Fig. S3

A schematic representation showing the gene structure of the six wheat MYB TFs investigated in this study. The positions of introns and exons, represented by lines and boxes, respectively, are indicated in each gene.

Supplementary Table S1. List of primers used in this study. The directional TOPO cloning overhang CACC, restriction enzyme sites and protection nucleotides are in bold.

**Primer purposes**	**Genes (CDS) or promoters**	**Forward primer**	**Reverse primer**
Cloning MYB TFs	*TaMYB16*	TTCGTTCGTGGGAGCGTTAG	CAAAGCATGTGCAGAGGTCG
		**CACC**ATGGGGCGATCGCCGTGCT	TCAGAACTCTGGCGCCG
	TaMYB24	CAGTCCCCTCTCCTCACCTC	GTTAGGTGGGCATGCAGTGA
		**CACC**ATGGGGAGGCCGCCGTGCT	CTAGAAAGGGTAGCCCAGG
	TaMYB31	TGTGCCTAGCCAGCCAAG	CCCAGCTCGATCTAAATCACC
		**CACC**ATGGGGAGGCCTCCGTGCT	TTAGAAGAACTCACTGGGGTC
	TaMYB74	ACTCCAGCTGCGAGACAAAC	CTCGGTCGGTAGTACGTGATG
		**CACC**ATGGGGCGCGCGCCGTG	CTACATGTAGTCGCTCACATCCAG
	TaMYB77	GCAGCATATTACGCCACTCC	CGACCTGTCGATGAAGCAG
		**CACC**ATGGGGCGATCACCATG	CTAGAGAAATGCTGGTGG
	TaMYB78	GTATACAAGGGCCGCCATG	ACACCTGCTTTGCAATGGG
		**CACC**ATGGGGAGGGCGCCGTGCT	TCAGCACGCGTCGGAGAG
Cloning promoters	*TaKCS1*	GTACGAAATCTCTCCAAGTCTTCC	GGATCTTGACGATGATGCTGG
		**CACC**CCAAGTCTTCCCCATGC	GGATCTCGAGACGTACG
	TaATT1	CAGACAAATGTTACATGCGGAG	GATCCACTCGTGCATGTCCTC
		**CACC**GGGTACTAGAGAAGAGAGCCATG	TGCCGGCCTCCCTG
	TdSHN1	**CACC**ATGGTGCAACCCAAGAAGAAGTTCC	TCAGACGACGAAGCTACCTTCTTCTCCA
		ACCTGCCTTCGCCTTGACAC	GCTCAGCAGCTCCTCGATCA
		**CACC**ATCCACCATCTCAGCCAAAATAC	GGAGGCAGAAGACAAGAGCGAGAT
Gene expression	*TaMYB16*	GACAGAGGAGGAGAAGAACTAC	GTCGCCAGCACTCAGAAC
	TaMYB24	ACCGTGCCAAGTTATCAAGG	TAAGTAACACAGGAGACCAAGG
	TaMYB31	TGGAGAACTGGCTGCTTG	CGTACTTAGAAGAACTCACTGG
	TaMYB74	CAGATGCTCCTCCCTTGG	GTGATCCTGGTGTAGTTGC
	TaMYB77	ACCAACTTCAATCACTCTG	ATCGCTTCTCAACTTACAC
	TaMYB78	AGAAACAATAGCAAAGCAGGTG	CTCAGACGCCATATACGACTC
Yeast hybrid	*TaMYB16*	**GAAGAATTC**ATGGGGCGATCGCCGTGCTG	**GGAGGATCC**TCAGAACTCTGG
	TaMYB16D1	**GAAGAATTC**ATGGGGCGATCGCCGTGCTG	**GGAGGATCC**TCAAAGCAGCCCGGTGAAG
	TaMYB16D2	**GAAGAATTC**ATGGGGCGATCGCCGTGCTG	**GGAGGATCC**TCAGTACGCCTGCATGG

	TaMYB24	**GAAGAATTC**ATGGGGAGGCCGCCGTGCTG	**GGAGGATCC**CTAGAAAGGGTAGCCCAG
	TaMYB24D1	**GAAGAATTC**ATGGGGAGGCCGCCGTGCTG	**GGAGGATCC**TAGAACGCGGACCCCAGCGCAC
	TaMYB31	**GAAGAATTC**ATGGGGAGGCCTCC	**GGAGGATCC**TTAGAAGAACTCACTGG
	TaMYB31D1	**GAAGAATTC**ATGGGGAGGCCTCC	**GGAGGATCC**TTACAGCATGGAGAACG
	TaMYB31D2	**GAAGAATTC**ATGGGGAGGCCTCC	**GGAGGATCC**TTAGGGAGTCTGCGCTG
	TaMYB74	**GAAGAATTC**ATGGGGCGCGCGCC	**GGAGGATCC**CTACATGTAGTCGCTCACATCC
	TaMYB74D1	**GAAGAATTC**ATGGGGCGCGCGCC	**GGAGGATCC**TACGCGTGAACCAGG
	TaMYB77	**GAAGAATTC**ATGGGGCGATCACCATG	**GGAGGATCC**CTAGAGAAATGCTGGTGG
	TaMYB77D1	**GAAGAATTC**ATGGGGCGATCACCATG	**GGAGGATCC**CTAGCTTCTCAACTTACACG
	TaMYB78	**GAAGAATTC**ATGGGGAGGGCGCCGTGCTG	**GGAGGATCC**TCAGCACGCGTCGGAGAG
	TaMYB78D1	**GAAGAATTC**ATGGGGAGGGCGCCGTGCTG	**GGAGGATCC**TCATGCTGTAACGCTGCTGG
	TaMYB78D2	**GAAGAATTC**ATGGGGAGGGCGCCGTGCTG	**GGAGGATCC**TCAGAGGACACCAGTCTGATCAAC
TdSHN1 promoter deletions	*SHN1D1*	**CACC**GCTCAAGGCTTCTG	TTGTTCTGCCTGTC
	SHN1D2	**CACC**GTACCTGACCTGTTG	TTGTTCTGCCTGTC
	SHN1D3	**CACC**TCTCGGGATCTGATC	TTGTTCTGCCTGTC
	SHN1D4	**CACC**CACCGACAGTCCAC	TTGTTCTGCCTGTC
	SHN1D5	**CACC**GACTACCTACGCATC	TTGTTCTGCCTGTC
	SHN1D6	**CACC**GCAGAGGCAAGTAC	TTGTTCTGCCTGTC

Supplementary Table S2. Amino acid residues of TaMYB74 forming hydrogen bonds with 12-bp DNA *cis*-elements of MYBR1 (5'-AGGTGGTTATGC-3'/5'-GCATAACCACCT-3') and MYBR2 (5'-ATCTAACCACAT-3'/5'-ATGTGGTTAGAT-3'). Core binding sequences in *cis*-elements are underlined.

Residues	Number of hydrogen bonds with MYBR1 and their distances in Å[1]									DNA phospho-diester backbone	Number
	Sense strand (5'-3')							Antisense strand (5'-3')			
	T_4	G_5	G_6	T_7	T_8	A_9	T_{10}	$A_{6'}$	$C_{7'}$		
Lys13	-	-	-	-	-	-	-	-	-	2 (2.9; 3.3)	2
Lys14	-	-	-	1 (2.9)	-	-	-	-	-	-	1
Trp17	-	-	-	-	-	-	-	-	-	1 (3.0)	1
Arg48	-	-	-	-	-	-	-	-	-	1 (3.5)	1
Lys51	-	-	-	-	1 (3.1)	1 (3.1)	1 (2.9)	-	-		3
Arg54	-	-	-	-	-	-	-	-	-	1 (2.9)	1
Arg56	-	-	-	-	-	-	-	-	-	1 (3.0)	1
Asn87	-	-	-	-	-	-	-	-	-	1 (3.1)	1
Trp89	-	-	-	-	-	-	-	-	-	1 (2.8)	1
Ser90	-	-	-	-	-	-	-	-	-	1 (3.2)	1
Asn102	-	-	1 (3.5)	-	-	-	-	-	-	-	1
Lys105	-	-	1 (2.8)	-	-	-	-	1 (3.2)	1 (3.5)	-	3
Asn106	-	1 (3.0)	-	-	-	-	-	-	-	-	1
Asn109	1 (3.6)	-	-	-	-	-	-	-	-	-	1
Arg115	-	-	-	-	-	-	-	-	-	2 (2.8; 3.2)	2
Total	1	1	2	1	1	1	1	1	1	11	20

Residues	Number of hydrogen bonds with MYBR2 and their distances in Å[1]									DNA phospho-diester backbone	Number
	Antisense strand (5'-3')							Sense strand (5'-3')			
	$T_{4'}$	$G_{5'}$	$G_{6'}$	$T_{7'}$	$T_{8'}$	$A_{9'}$	$G_{10'}$	A_6	C_7		
Lys13	-	-	-	-	-	-	-	-	-	1 (2.8)	1
Lys14	-	-	1 (3.5)	-	-	-	-	-	-	-	1
Trp17	-	-	-	-	-	-	-	-	-	1 (2.9)	1
Arg48	-	-	-	-	-	-	-	-	-	-	-
Lys51	-	-	-	-	-	1 (3.3)	1 (3.0)	-	-	-	2
Arg54	-	-	-	-	-	-	-	-	-	1 (3.1)	1
Arg56	-	-	-	-	-	-	-	-	-	1 (2.8)	1
Asn87	-	-	-	-	-	-	-	-	-	1 (3.2)	1
Trp89	-	-	-	-	-	-	-	-	-	1 (2.8)	1
Ser90	-	-	-	-	-	-	-	-	-	1 (2.8)	1
Asn102	-	-	1 (3.3)	-	-	-	-	1 (3.6)	-	-	2
Lys105	-	-	1 (2.8)	-	-	-	-	1 (3.6)	1 (3.6)	-	3
Asn106	-	1 (3.0)	-	-	-	-	-	-	1 (3.4)	-	2
Asn109	-	-	-	-	-	-	-	-	-	-	-
Arg115	-	-	-	-	-	-	-	-	-	1 (2.8)	1
Total	-	1	3	-	-	1	1	2	2	8	18

[1]Separations equal to or less than 3.6 Å are indicated in brackets.
